# Identifying Biomarkers of Wharton’s Jelly Mesenchymal Stromal Cells Using a Dynamic Metabolic Model: The Cell Passage Effect

**DOI:** 10.3390/metabo8010018

**Published:** 2018-02-24

**Authors:** Benoît Laflaquière, Gabrielle Leclercq, Chandarong Choey, Jingkui Chen, Sabine Peres, Caryn Ito, Mario Jolicoeur

**Affiliations:** 1 Department of Chemical Engineering, Research Laboratory in Applied Metabolic Engineering, École Polytechnique de Montréal, C.P.6079, Centre-ville Station, Montréal, QC H3C 3A7, Canada; benoit.laflaquiere@hotmail.fr (B.L.); gabrielle.leclercq06@gmail.com (G.L.); jingkui.chen@polymtl.ca (J.C.); sabine.peres@lri.fr (S.P.); 2 Sprott Centre for Stem Cell Research, Ottawa Hospital Research Institute, 501 Smyth Rd. CCW 5105a, Ottawa, ON K1H 8L6, Canada; chandarongc@gmail.com (C.C.); cito@ohri.ca (C.I.); 3 LRI, Université Paris-Sud, CNRS, Université Paris-Saclay, 91405 Orsay, France; 4 MaIAGE, INRA, Université Paris-Saclay, 78350 Jouy-en-Josas, France

**Keywords:** metabolomics, Wharton’s Jelly mesenchymal stem/stromal cells (WJMSC), immunosuppression, biomarkers

## Abstract

Because of their unique ability to modulate the immune system, mesenchymal stromal cells (MSCs) are widely studied to develop cell therapies for detrimental immune and inflammatory disorders. However, controlling the final cell phenotype and determining immunosuppressive function following cell amplification in vitro often requires prolonged cell culture assays, all of which contribute to major bottlenecks, limiting the clinical emergence of cell therapies. For instance, the multipotent Wharton’s Jelly mesenchymal stem/stromal cells (WJMSC), extracted from human umbilical cord, exhibit immunosuppressive traits under pro-inflammatory conditions, in the presence of interferon-γ (IFNγ), and tumor necrosis factor-α (TNFα). However, WJMSCs require co-culture bioassays with immune cells, which can take days, to confirm their immunomodulatory function. Therefore, the establishment of robust cell therapies would benefit from fast and reliable characterization assays. To this end, we have explored the metabolic behaviour of WJMSCs in in vitro culture, to identify biomarkers that are specific to the cell passage effect and the loss of their immunosuppressive phenotype. We clearly show distinct metabolic behaviours comparing WJMSCs at the fourth (P4) and the late ninth (P9) passages, although both P4 and P9 cells do not exhibit significant differences in their low immunosuppressive capacity. Metabolomics data were analysed using an in silico modelling platform specifically adapted to WJMSCs. Of interest, P4 cells exhibit a glycolytic metabolism compared to late passage (P9) cells, which show a phosphorylation oxidative metabolism, while P4 cells show a doubling time of 29 h representing almost half of that for P9 cells (46 h). We also clearly show that fourth passage WJMSCs still express known immunosuppressive biomarkers, although, this behaviour shows overlapping with a senescence phenotype.

## 1. Introduction

Immunosuppressive cells play crucial roles in regulating the human immune system, by secreting pro- and anti-inflammatory cytokines at ratio enabling desired specific immune response. A dysfunction of this equilibrium can lead to serious pathological disorders. For instance, the overexpression of pro-inflammatory cytokines has been identified as a main cause leading to Multiple Sclerosis [1] and graft-versus-host diseases [2]. Conversely, the overexpression of anti-inflammatory cytokines results in a deficient immune system unable to eliminate a pathogen or tumour cells [3].

Indeed, several cell types revealed a functional capacity to impact on this cytokine equilibrium, either by directly increasing or reducing their respective concentrations or by modulating the production or consumption rates of specific metabolites within the cell microenvironment, that indirectly affects cytokines secretion. For instance, macrophages exhibit specific behaviour depending on whether they are polarized towards the M1 or M2 phase [4]. In the M2 phase, an increased consumption rate of tryptophan is observed, which leads to the starvation of the surrounding T-cells [5,6]. Also, recruited by tumor cells, myeloid derived suppressor cells (MDSC) are known to protect the tumor against the immune system [7]. Once recruited, MDSCs increase their consumption in l-arginine (L-ARG) and their production of nitric-oxide (NO), both phenomenon resulting in reduced T-cell proliferation and viability [8,9]. Interestingly, mesenchymal stem/stromal cells (MSC) placed within a pro-inflammatory environment appear to reduce the immune response [10]. For instance, bone marrow mesenchymal stem/stromal cells (BMMSCs), as induced by the pro-inflammatory cytokine interferon-γ (IFNγ), increase their uptake of tryptophan resulting in reduced T-cells responses [11]. More recently, Wharton’s Jelly mesenchymal stem cells (WJMSC) when compared to bone marrow derived MSCs [12], have revealed strong immunosuppressive properties under pro-inflammatory conditions with INFγ and tumor necrosis factor-α (TNFα) [13,14]. Moreover, MSCs do not express Human Leukocyte Antigen—antigen D Related (HLA-DR) or co-stimulatory clusters of differentiation 40 (CD40), CD40L, CD80, and CD86 cell surface antigens necessary to trigger an immune response [15,16,17]. Therefore, their low immunogenicity evades immune recognition upon xeno or allogeneic transplantation. Thus, the unique MSCs immunomodulatory properties have attracted the interest of the community resulting in nearly 200 clinical trials so far to test their efficacy at modulating severe immune and inflammatory responses associated to various medical pathologies [18]. However, data emerging from these trials and preclinical studies are often inconsistent and contradictory [18,19]. Besides pointing out the inherent disparities between MSC donors and tissue sources [20,21], most of the compelling cause of these varying results may be attributed to the in vitro processes to isolate and amplify MSCs. Indeed, MSCs immunomodulatory function has been shown to decrease in prolonged in vitro culture, reaching undetectable levels at late passages [22,23].

MSCs are a heterogeneous population and the International Society for Cell Therapy recognizes the need for standardized metrics to assess MSC immunoregulatory function to enable clinical development [24,25]. The problem with the current metrics is that they are too broad and impractical to rapidly determine cell phenotype in time for a clinician to decide whether or not to proceed with the injection of MSCs. Currently, cell culture assays require days to confirm MSC effects on T-cell or macrophage growth and function. However, “omics” approaches clearly open opportunities for the identification of efficient biomarkers correlating with cell function to provide fast and reliable assays. Indeed, metabolomics is gaining in interest since it allows an integrated and functional view on a cell population behaviour, with the genomic and the transcriptomics in action [26]. For instance, only few metabolites are known to be involved in cell immunosuppressive capacity [6,8,27], and the link between their concentration and cells phenotype is still not clear. Therefore, the identification of biomarkers of MSCs immunosuppressive phenotype may provide insight into their metabolic network.

Here, we present a study on the metabolic behaviour of Wharton’s Jelly mesenchymal stem cells (WJMSC), when comparing fourth (P4) to late ninth passage (P9) cells, wherein both cells presented slightly different immunosuppressive function under pro-inflammatory conditions. A dynamic metabolic flux analysis (dMFA) was performed to further characterize differences in the behaviour of both WJMSC cultures, using an in silico platform describing central carbon metabolism as well as pathways related to cell immunomodulatory mechanisms [28]. A dynamic metabolic model was developed based on the experimental data of intracellular and extracellular metabolite concentrations. This approach is complementary to steady-state approaches [29] with the prediction of extracellular and intracellular metabolite concentrations, as well as metabolic flux distribution.

## 2. Results and Discussion

### 2.1. Fourth (P4) and Ninth (P9) Passage WJMSC Cells Exhibit Non-Significantly Different Low Immunosuppressive Phenotypes

**Figure 1:**
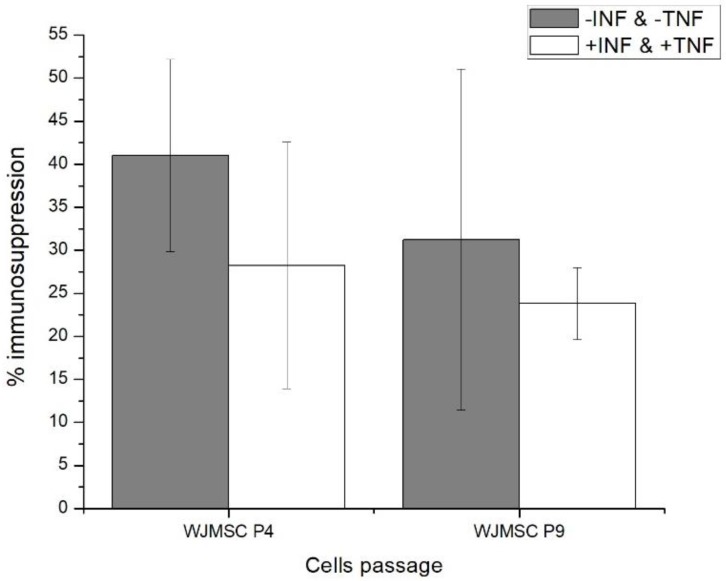
Mixed lymphocyte reaction (MLR) data of Wharton’s Jelly mesenchymal stem cells (WJMSC) cells.

A mixed lymphocyte reaction analysis (MLR) was performed, as proposed by Weiss et al. [23], who revealed that the immunosuppressive phenotype of human umbilical cord mesenchymal stem cells (hUCMSC) strongly decreases between the 5th and the 9th passages. In the present case, MLR analysis of WJMSCs at the 4th and 9th passages show no significant difference in their immunomodulatory function, based on a Student’s *t*-test of 0.295 as compared to the reference value of 2.132 for the 95% confidence interval. However, although not statistically significant, one can observe a decreasing tendency of the cells immunomodulatory function between the 4th and the 9th passages (Figure 1). This observation suggests that similar to the work of Weiss et al. [19], WJMSCs may preserve strong immunomodulatory function among early passages, such as up to the 2nd or the 3rd. Of interest, similar conclusions can be drawn in the presence or absence of INFγ and TNFα. In addition, it should be noted that the large SEM error bars can be attributed to the fact that WJMSCs are a subset of hUCMSCs, which are composed of various types of MSCs that are found in the umbilical cord.

Therefore, since both P4 and P9 cells do not significantly differ in their low immunosuppressive function under pro-inflammatory conditions (i.e., +IFN & +TNF), a dynamic metabolomic study was performed to specifically characterize and document biomarkers that are specific to the cumulative effect of successive passages.

### 2.2. WJMSC P4 and P9 Cells Show Distinct Metabolic Behaviour

**Figure 2:**
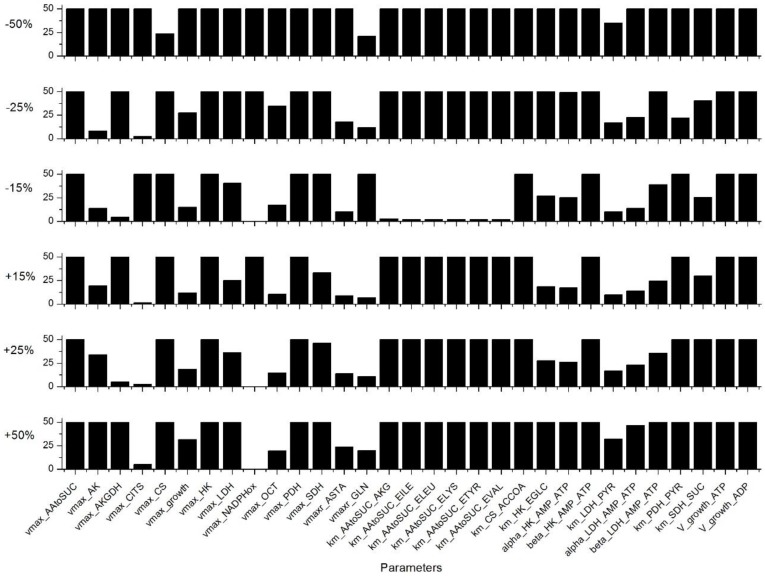
Sensitivity analysis results for the 31 most sensitive parameters. This selection is based on a simulation error criterion higher than 10% for a parameter value variation of ±15%. Simulation error higher than 50% are truncated.

The metabolic model was first calibrated combining experimental data for P4 and P9 cells, as described in Appendix B. The value of the 234 parameters (Table S3, Supplementary Materials) that are minimizing model simulation error were then determined. Then, a sensitivity analysis was performed, assessing each parameter and the most sensitive parameters were identified as described in the Appendix C. Indeed, of the 234 parameters, 31 are considered as highly sensitive, as they induce a variation of more than 10% of the global simulation error from a change of only 15% of their value (Figure 2). Most of these parameters are involved in the TCA cycle: such as the theoretical asymptotic maximal activities $vmax_{AAtoSUC},$ $vmax_{AKGDH}$, $vmax_{CITS}$, $vmax_{CS}$, $vmax_{PDH}$, $vmax_{SDH}$, $vmaxr_{ASTA}$, and the half-saturation constants $Km_{AAtoSUC_{AKG}},$ $Km_{AAtoSUC_{ILE}},$ $Km_{AAtoSUC_{ELEU}}$, $Km_{AAtoSUC_{ELYS}}$, $Km_{AAtoSUC_{ETYR}}$, $Km_{AAtoSUC_{EVAL}}$, $Km_{CS_{ACCOA}}$, $Km_{PDH_{PYR}}$, and $Km_{SDH_{SUC}}$. Another group of sensitive model parameters is within the glycolysis pathway with especially reactions catalysed by HK and LDH with $vmax_{HK}$, $vmax_{LDH}$, $Km_{HK_{EGLC}}$, $alpha_{HK_{AMP_{ATP}}}$, $Km_{LDH_{PYR}}$, $beta_{HK_{AMP_{ATP}}}$, $alpha_{LDH_{AMP_{ATP}}}$, and $beta_{LDH_{AMP_{ATP}}}$. One parameter affects the glutaminolysis ($vmaxr_{GLN}$), one is involved in the urea cycle ($vmax_{OCT}$), and the others are related to cell energetics state and the cell specific growth rate with $vmax_{AK}$, $vmax_{NADPHox}$, $vgrowth_{ATP}$, $vgrowth_{ADP}$, and $vmax_{growth}$, respectively. Our results clearly show that model sensitive parameters are widely distributed throughout the metabolic network, and this is not surprising as it highlights that the central carbon metabolic pathways are vital when describing cell behaviour. Indeed, we have identified a similar subset of sensitive parameters in previous studies with CHO cells [28,30,31]. The sensitive role of the LDH enzyme has also been identified by Nolan et al. for another CHO cell line [32]. Interestingly, the parameters of the intermediate reactions of glycolysis, such as PGI, phosphoglycerate kinase (PGK), or PK were not sensitive in the WJMSC model, contrarily to CHO cells [28,30,31].

A first set of parameters were thus determined from minimizing simulation error, anchoring model structure and parameters on averaged WJMSCs behaviour combining P4 and P9 cell culture data sets. Then, a parameter sensitivity analysis to allow the specific identification of 31 highly sensitive parameters; a parameters subset enabling us to distinctively describe P4 and P9 cell behaviours in a second step, as shown in a previous work [28], keeping constant the less sensitive parameters in exception of $vmax_{resp}$ (Table 1), which was also modified for its direct high impact on cell energetics (e.g., ATP-to-ADP ratio). Of interest, it can be noticed that for eight parameters (of 32), i.e., $vmax_{AK}$, $vmaxr_{ASTA}$, $vmax_{CITS}$, $vmax_{CS}$, $vmax_{LDH}$, $Km_{LDH_{PYR}}$, $km_{AAtoSUC_{EILE}}$, and $alpha_{LDH_{AMP_{ATP}}}$, optimal values only differed by less than 15% when distinctively adapting the model to P4 and P9 cells. However, the other 24 sensitive parameters (including $vmax_{resp}$) needed to be significantly adjusted in the process of model adaptation. Therefore, only 24 parameter values needed to be modified to allow adapting of a 234 parameter model to either P4 and P9 cells. Differences in model parameter values between P4 and P9 cells, presented as supplementary data, were useful to further characterize the changes in behaviour comparing P4 and P9 cells, as discussed in the following sections. Model parameters were thus determined to minimize the global error between model simulations and experimental data. Therefore, the model simulations represent the optimal estimates of cells and metabolites concentration with time, considering the simplified reaction network under study. Also, it is important to note that all the model simulation curves shown in all figures were extracted from a unique time-solution of the model, and thus can be seen as occurring concurrently.

### 2.3. P4 Cells Present a Faster Doubling Time than Late P9 Passage Cells

**Figure 3:**
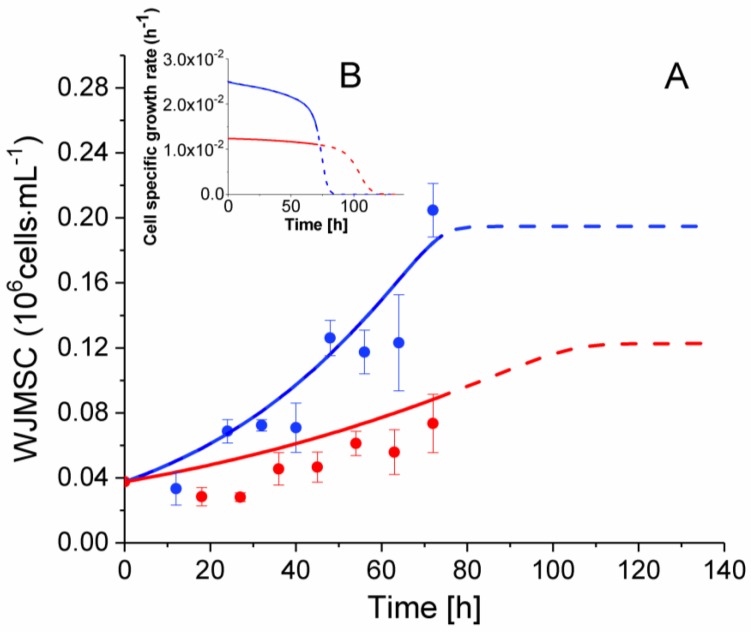
Effect of cell passage on cell growth. (**A**) WJMSC cell population with time. (**B**) Insert graph presents model simulations of WJMSCs specific growth rates. ● P4 WJMSC experimental data, ● P9 WJMSC experimental data. Blue lines are for P4 cell simulations and red lines are for P9 cell simulation, with dashed lines indicating model extrapolations over the time of harvest of the cultures. Average data with Standard Error of the Mean (SEM) are shown for *n* = 3.

Cultivated under inflammatory conditions from 0 to 72 h, P4 WJMSC cells show a higher specific average growth rate of 2.36 × 10^−2^ h^−1^ (i.e., doubling time of 29.4 h) as compared to 1.36 × 10^−2^ h^−1^ (i.e., doubling time of 51.0 h) for the late P9 passage cells (Figure 3). Indeed, this difference in their growth rate is also captured in the model, with a maximum specific growth rate ($vmax_{growth}$) for P4 cells of two times that of P9 cells (Table 1). From a similar inoculation of 0.0375 × 10^6^ cells mL^−1^, P4 cells reached 0.205 ± 0.029 × 10^6^ cells mL^−1^ at 72 h as compared to 0.1 ± 0.026 × 10^6^ cells mL^−1^ for P9 cells. This result is in agreement with Kang et al. who observed a decreasing growth rate with an increase of the passage number in human bone marrow mesenchymal stem cells and umbilical cord blood cells [33]. Both of the cell cultures were stopped at 72 h here, when P4 cells reached confluency. Behavioral artifacts are generated when the cells reach confluency with dense cell-cell contacts.

Interestingly, model simulations, which cope with both P4 and P9 cell growth trends, allow for further analysing the effect of the cell passage number on WJMSC cells. Indeed, it was first intriguing to question the model for potential limiting nutrients that could have limited the culture post-confluency since cell cultures were both simultaneously stopped when P4 reached confluency at 72 h. Model simulations were thus prolonged from 72 h until simulating growth cessations in both cultures, under speculative prolonged cultures with no cell confluency phenomena (model extrapolations are indicated as dashed lines in all figures). It was also possible to plot the cell specific growth rate with time (Figure 3B). Values available from the same model simulations are shown in Figure 3. The model thus estimates an initial specific growth rate of 2.5 × 10^−2^ h^−1^ after inoculation for P4 cells; a rate that continuously decreases until 60 h (µ of 2.1 × 10^−2^ h^−1^), then rapidly reaches growth cessation at 85 h. However, the specific growth rate for P9 cells starts at 1.24 × 10^−2^ h^−1^, and then continuously decreases until 90 h (µ of 2.1 × 10^−3^ h^−1^), rapidly reaching growth cessation at 120 h. As indicated in Table 2, the specific growth rates estimated by the model were similar to the values calculated from experimental data between 0 and 72 h for P4 and P9 cells. The nutritional limitation phenomenon, which is normally expected to cause growth arrest, has thus been addressed, and the amino acid tryptophan has been identified as the most probable limiting nutrient from model simulation and experimental results presented in Section 2.8 below.

### 2.4. P4 Cells Present a More Active Metabolism Than P9 Passage Cells

#### 2.4.1. Glycolysis Pathway

**Figure 4:**
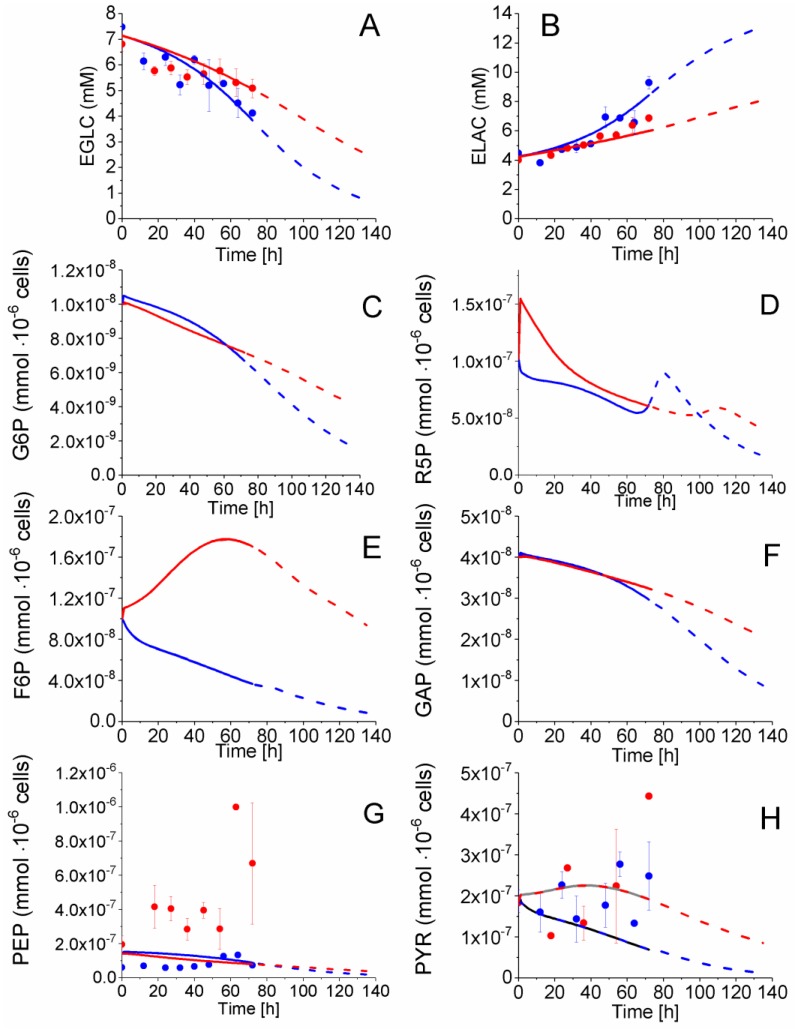
Effect of cell passage on glycolysis and pentose phosphate pathways intermediates concentration. (**A**) Extracellular glucose concentration; (**B**) Extracellular lactate concentration; (**C**) Glucose-6-phosphate concentration; (**D**) Ribose-5-phosphate concentration; (**E**) Fructose-6-phosphate concentration; (**F**) Glyceraldehyde-3-phosphate concentration; (**G**) Phosphoenolpyruvate concentration; and, (**H**) Pyruvate concentration. Same conditions and symbols as in Figure 3 are applied.

Extracellular glucose (EGLC) uptake, extracellular lactate (ELAC) production, and intracellular pyruvate (PYR), and phosphoenolpyruvate (PEP) concentrations were measured with time whereas glucose-6-phosphate (G6P), fructose-6-phosphate (F6P) and glyceraldehyde-3-phosphate (GAP) concentrations were estimated from model simulations (Figure 4). Before 54 h, glucose consumption and lactate secretion behaviour followed similar trends for both P4 and P9 cells, calculated from experimental data and model simulation. However, according to model simulation of species concentration, the cell passage effect starts being observed from 54 h with EGLC and ELAC concentrations showing diverging trends comparing P4 to P9 cells. Indeed, from 54 to 72 h, an average specific glucose uptake rate of 3.4 × 10^−4^ mmol·10^−6^ cells·h^−1^ was estimated for P4 cells as compared to 2.4 × 10^−4^ mmol·10^−6^ cells·h^−1^ for P9 cells, whereas the average lactate specific production rates were of 5.8 × 10^−4^ mmol·10^−6^ cells·h^−1^ for P4 cells and 5.6 × 10^−4^ mmol·10^−6^ cells·h^−1^, according to experimental data. Looking at intracellular metabolic intermediates of glycolysis, PYR concentration remained constant and at similar levels of 2 × 10^−7^ mmol·10^−6^ cells in P4 and P9 cells (Figure 4H), while PEP cell concentration also remained constant but at distinct levels with 1 × 10^−7^ mmol·10^−6^ cells in P4 cells and 4 × 10^−7^ mmol·10^−6^ cells in P9 cells (Figure 4G).

**Figure 5:**
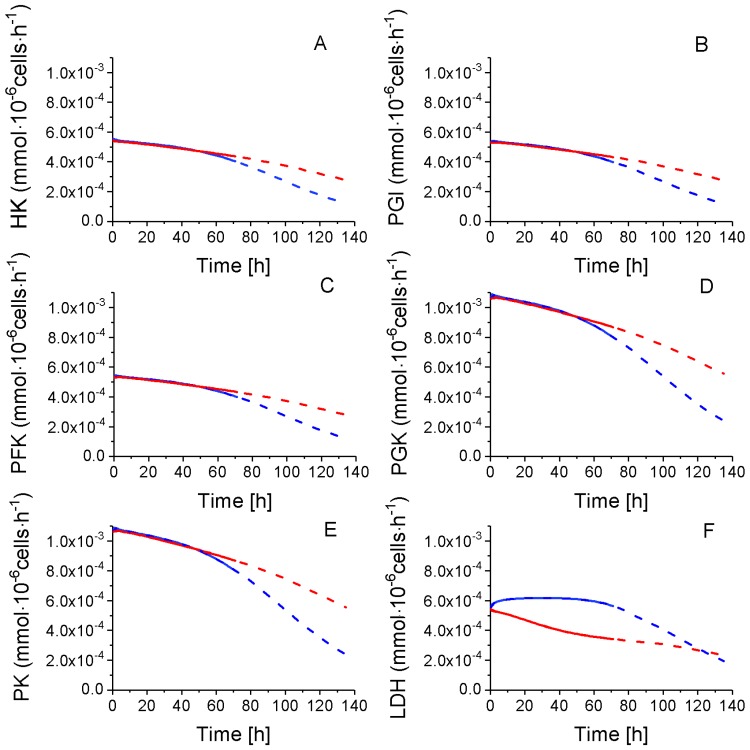
Glycolysis fluxes of WJMSC. (**A**) Hexokinase flux; (**B**) Phosphoglucose isomerase flux; (**C**) Phosphofructokinase flux; (**D**) Phosphoglycerate kinase flux; (**E**) Pyruvate kinase flux; and, (**F**) Lactate dehydrogenase flux. Same conditions and symbols as in Figure 3 applied.

As mentioned above, a total of eight sensitive model parameters are part of glycolysis (Table 1) and a subset of these required modification while adapting the model to P4 and P9: $vmax_{HK}$ (×0.5), $km_{HK_{EGLC}}$ (×1.22), $alpha_{HK_{AMP_{ATP}}}$ (×1×1.22), $beta_{HK_{AMP_{ATP}}}$ (×1×1.22), $vmax_{LDH}$ (idem), $km_{LDH_{PYR}}$ (idem), $alpha_{LDH_{AMP_{AT}}}$ (idem) and $beta_{LDH_{AMP_{AT}}}$ (×0.86). Of interest, all of these parameters are exclusively related to the entry (HK) and the major output (LDH) of glycolysis. Despite P9 cells having a $vmax_{HK}$ reduced by 50% (Table 1), all of the simulated glycolytic fluxes are similar to those for P9 cells the first 54 h (Figure 5), from which a shift is observed in culture behaviour; a result which is clearly suggesting the primary role of cell energetics on flux regulation (Table S5, Supplementary Materials). Globally, glycolysis shows similar concentration behaviour from EGLC to PYR in P4 and P9 cells before 54 h, while the model simulates constantly decreasing fluxes, except for LDH, which stayed stable at high levels in P4 cells, concurrent with cell growth. Thus, except for LDH, all other glycolysis fluxes show diverging trends with a more pronounced decrease in P4 as compared to P9 cells. P4 cells show a lower specific (i.e., normalized per 10^6^ cells) glucose uptake rate than P9 cells after 54 h, but a higher specific lactate production rate during the whole culture. However, due to their higher specific cell growth rate, P4 cells culture lead to a higher final decrease of glucose concentration in the culture medium. Thus, P4 cells show a more active glycolytic metabolism during the fast growth period (0–54 h) with a similar specific glucose uptake rate, while a higher lactate specific production rate. P4 and P9 WJMSC cells present a glucose uptake and a lactate production rates before 54 h that are twice those reported for CHO cells. Indeed, our results agree with those of Moya et al. [34] who observed a glycolytic metabolism in human MSCs (hMSC). Higher glycolysis was also observed when hMSC were plated at low densities versus higher densities, with lactate production to glucose consumption rates ratios of ~3 and ~1.8, respectively [35]. Higher ratios than 2 are attributed to the contribution of some amino acid metabolism, such as of glutamine in lactate production [36]. Furthermore, CHO cells show a glucose uptake rate of 1–2 × 10^−4^ mmol·10^−6^ cells·h^−1^ and a lactate production rate of ~2 × 10^−4^ mmol·10^−6^ cells·h^−1^, according to our previous work [28,31] or to other studies [32,37,38].

**Figure 6:**
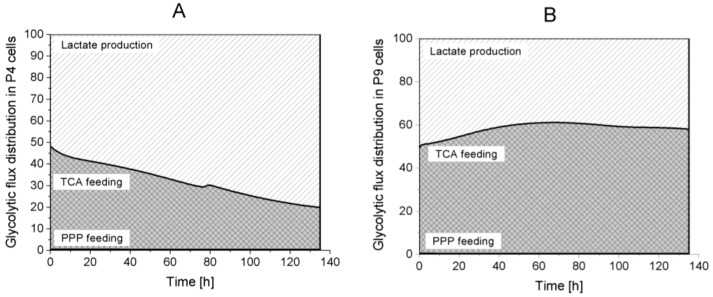
Glycolysis flux distribution. (**A**) P4 cell culture; (**B**) P9 cell culture. 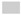 Lactate production, 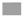 TCA feeding, 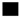 pentose phosphate pathways (PPP) feeding.

Model simulations suggest that most of the glycolytic flux feeds pyruvate synthesis. Indeed, the pentose phosphate pathway (PPP) accounts for less than 1% of the total glycolytic flux (Figure 6). From pyruvate, the metabolic flux is distributed either to lactate or to the TCA cycle. Interestingly, P4 cells direct more than 50% of their glycolytic flux to lactate production during the entire culture, whereas P9 cells show opposite behaviour with more than 50% of glycolytic flux feeding to the TCA cycle. These results highlight that P4 cells mainly have a glycolytic metabolism compared to P9 cells, which show an oxidative phosphorylation metabolism. P9 cells may also be undergoing senescence [35]. Of interest, model simulation results suggest that P9 cells have a higher demand on energy. This possible switch from a glycolytic to a respiratory metabolism may represent a valuable biomarker of the phenotypic transition that seem to occur with increasing cell passages. However, although the literature clearly identified a aerobic glycolytic metabolism as a character of immunosuppressive cells [39], further characterization of immunosuppressive WJMSCs is still required. Our results may simply confirm that P4 cells still express (but not significantly) immunosuppressive traits when compared to P9 cells.

#### 2.4.2. The Pentose Phosphate Pathway Is More Solicited in P9 Cells

**Figure 7:**
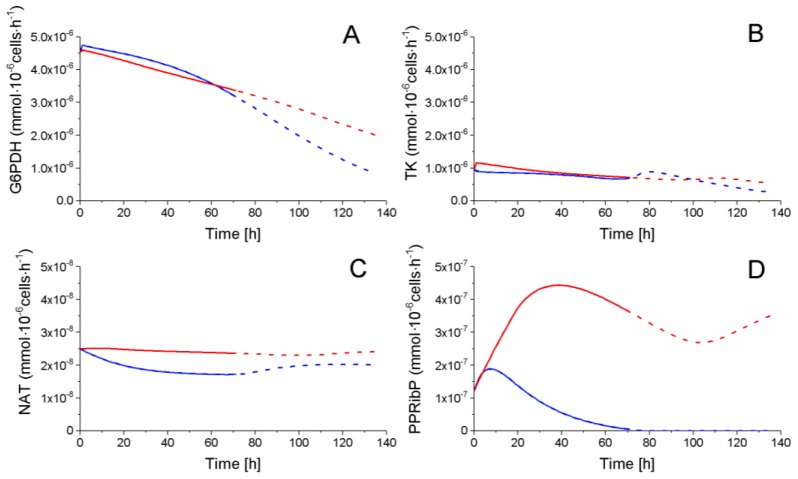
Fluxes of PPP. (**A**) Glucose-6-phosphate dehydrogenase flux; (**B**) Transketolase flux; (**C**) NAT flux; and, (**D**) Phosphoribosyl pyrophosphate flux. Same conditions and symbols as in Figure 3 are applied.

The pentose phosphate pathways (PPP) is consuming a minor part of the glycolytic flux, as mentioned above, with a glucose-6-phosphate dehydrogenase (G6PDH) flux of ~10^−6^ mmol·10^−6^ cells·h^−1^ as compared to a glycolytic global flux of ~10^−4^ mmol·10^−6^ cells·h^−1^ for both P4 and P9 cells (Figure 7). Ribose-5-phosphate (R5P), the only PPP intermediate, which cell concentration was quantified, shows a similar decreasing trend for both P4 and P9 cells (Figure 4). The transketolase (TK) enzyme shows a similar decreasing trend for both P4 and P9 cells, and the reaction flux producing NAD from R5P (NAT) stayed constant for both conditions with, however, a higher value for P9 cells. Interestingly, phosphoribosyl pyrophosphate (PPRibP) activity is much higher in P9 cells with 2.5 × 10^−7^ mmol·10^−6^ cells·h^−1^, whereas it decreases in P4 cells at 80 h from an initial value of 12 × 10^−7^ to 1.7 × 10^−1^ mmol·10^−6^ cells·h^−1^. Of interest, the simulated G6PDH flux is similar in magnitude (~10^−6^ mmol·10^−6^ cells·h^−1^) to values reported in literature for CHO cells [31,37].

**Figure 8:**
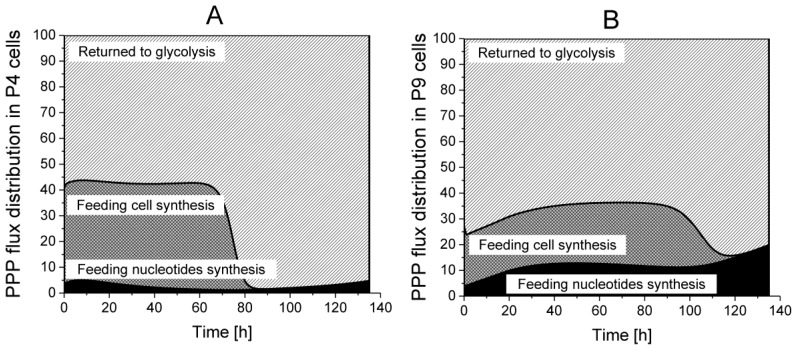
Flux distribution in PPP. (**A**) P4 cell culture; (**B**) P9 cell culture. 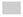 Returned to Glycolysis, 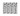 Feeding cell synthesis, 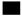 Feeding nucleotides synthesis.

The analysis, by the use of model simulation, regarding the distribution of PPP fluxes to cell synthesis, nucleotides synthesis as well as returning to glycolysis, reveals that 55% of the incoming G6PDH flux returns to glycolysis in P4 cells, but this contribution reaches 65 to 75% in P9 cells, in their growth phase (Figure 8). However, P4 cells direct a higher part of the PPP flux to cell synthesis as compared to P9 cells; a result agreeing with P4 higher specific cell growth rate observed experimentally. P4 cells require around 40% of the PPP flux in their growth phase, whereas it reaches only 20% in P9 cells. Furthermore, P4 cells only need 3 to 5% of the PPP flux for nucleotide synthesis, which represents half of P9 cells requirements. PPP flux dynamics maintained a NADPH-to-NADP ratio of ~1 (Figure S1), which was similar to that observed in hMSCs [35].

### 2.5. P9 Cells Maintain a Higher TCA Activity

**Figure 9:**
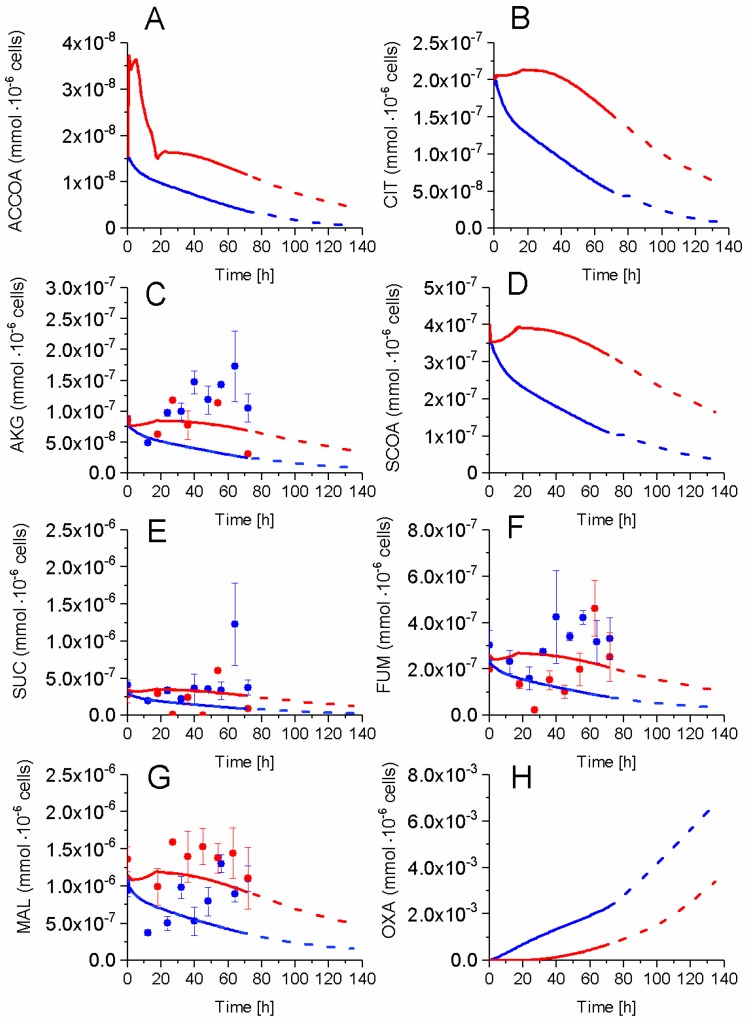
Effect of cell passage on TCA cycle intermediates concentration. (**A**) Acetyl-CoA concentration; (**B**) Citrate concentration; (**C**) Alpha-ketoglutarate concentration; (**D**) Succinyl-CoA concentration; (**E**) Succinate concentration; (**F**) Fumarate concentration; (**G**) Malate concentration; and, (**H**) Oxaloacetate concentration Same conditions and symbols as in Figure 3 are applied.

The impact of successive cell passages on TCA cycle activity was also studied. The cell concentrations in major TCA intermediates, such as malate (MAL), succinate (SUC), alpha-ketoglutarate (AKG), and fumarate (FUM) were measured while model simulation enabled the determination of citrate (CIT), succinyl-CoA (SCOA), oxaloacetate (OXA) concentrations, as well as the TCA precursor acetyl-CoA (ACCOA) (Figure 9). SUC and AKG concentrations showed similar but specific behaviours in P4 and P9 cells. SUC concentration remained constant in both cultures at an average value of 3.3 × 10^−7^ mmol·10^−6^ cells·h^−1^, increasing briefly from 54 h in P4 cells. In both conditions, AKG increased from 8.6 × 10^−8^ to 1.3 × 10^−7^ mmol·10^−6^ cells·h^−1^ at 54 h decreasing slightly thereafter. FUM concentration showed a similar increasing behaviour in both P4 and P9 cells, although maintaining a higher concentration in P4 cells. In the case of MAL, its intracellular concentration stayed higher in P9 as compared to P4 cells. In the simplified metabolic network described in the model, FUM is synthesized by succinate dehydrogenase (SDH) and also by argininosuccinate lyase (ASL), a combination that may explain a higher FUM concentration level in P4 cells due to a higher activity of ASL in the Urea cycle in P4 when compared to P9 cells. The concentration of non-measured metabolites were estimated from model simulation, with higher concentrations for all simulated TCA intermediates in P9 cells except for OXA. Higher amounts of TCA intermediates, which suggests a more active TCA cycle in P9 cells, agrees with the above-mentioned observation of a respiratory metabolism in these late passage P9 cells as compared to P4 cells.

**Figure 10:**
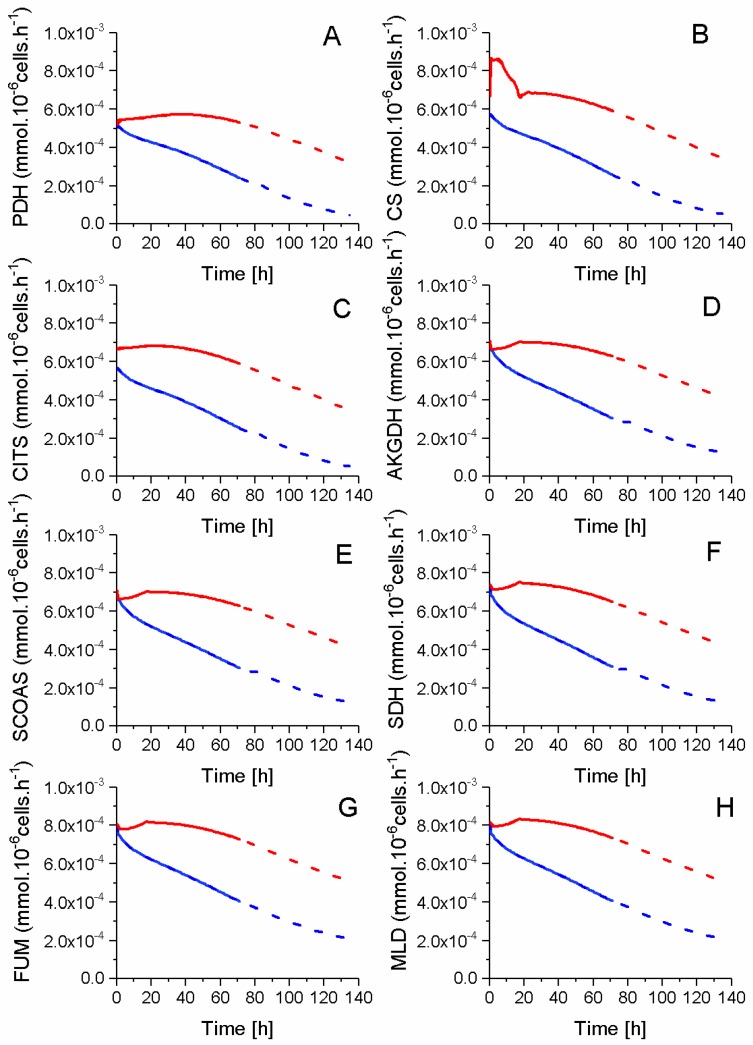
TCA intermediates flux profile. (**A**) Pyruvate dehydrogenase flux; (**B**) Citrate synthase flux; (**C**) aconitase/isocitrate dehydrogenase flux; (**D**) Alpha ketoglutarate dehydrogenase flux; (**E**) Succinyl-CoA synthetase flux; (**F**) Succinate dehydrogenase flux; and, (**G**) Fumarase flux; (**H**) Malate dehydrogenase flux. Same conditions and symbols as in Figure 3 are applied.

Model simulated fluxes coming from glycolysis are of a magnitude of 10^−4^ mmol·10^−6^ cells·h^−1^, whereas TCA metabolic intermediates show a concentration magnitude of ~10^−7^ mmol·10^−6^ cells (Figure 10). Therefore, a slight variation of a unique reaction flux can result in a high divergence of associated metabolite concentrations. Thus, this may explain the high sensitivity of model parameters involved in six TCA cycle reactions, such as pyruvate dehydrogenase (PDH), citrate synthase (CS), aconitase/isocitrate dehydrogenase (CITS), SDH, ASTA, and AAtoSUC. Interestingly, the changes in sensitive parameter values between P4 and P9 cells differ with the enzyme (Table 1). Indeed, AAtoSUC enzyme has the most modified kinetics, with a maximal flux rate augmenting by a factor of 22× and its global affinity constant being augmented by a factor of up to 3 (Table 1). Conversely, CS and CITS enzymes kinetics show the smallest variation with only ×1.2 of their maximum reaction rate, and ASTA and SDH enzymes did not require any modification of their parameter values. Overall, all the model simulated fluxes of TCA show higher values in P9 cells as compared to P4 cells. This result can be attributed to the higher entry flux from pyruvate to the TCA and PDH enzyme in P9 cells, as well as the non-accumulation of TCA intermediates in model simulations. However, the higher TCA activity of P9 cells did not lead to a higher growth rate compared to P4 cells, but most probably supported higher energy production and maintenance requirements.

### 2.6. P9 Cells Exhibit a Higher ATP Turnover Rate than P4 Cells

**Figure 11:**
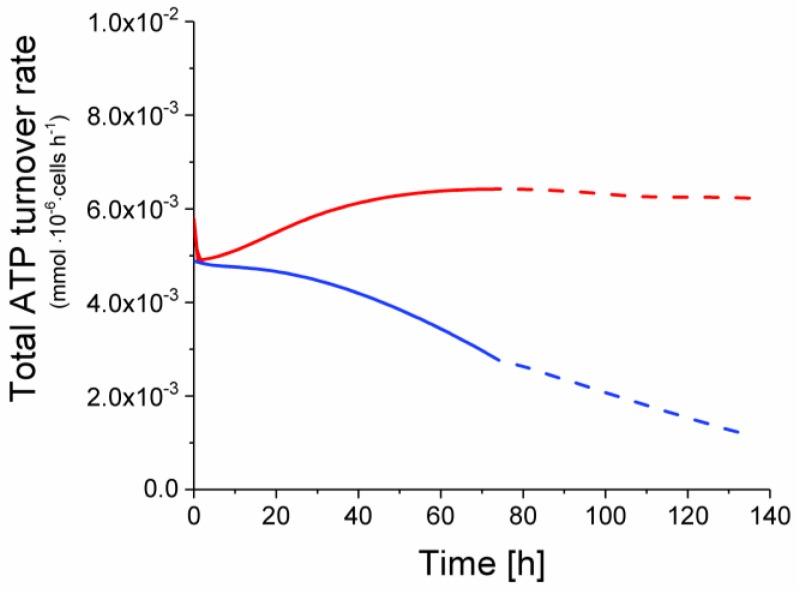
Total ATP turnover rate of early and late passage cells. Same conditions and symbols as in Figure 3 are applied.

Model simulation also allowed estimating the global ATP turnover rate. In agreement with an active TCA cycle, it is clear that the ATP turnover rate was higher in P9 cells, with a total ATP production rate starting from 4.9 × 10^−3^ and increasing at a stable value of 6.2 × 10^−3^ mmol·10^−6^ cells·h^−1^ (Figure 11). Conversely, the ATP turnover rate in P4 cells regularly decreased from an initial value of 4.9 × 10^−3^ to 1.2 × 10^−3^ mmol·10^−6^ cells·h^−1^ at the end of the simulation.

**Figure 12:**
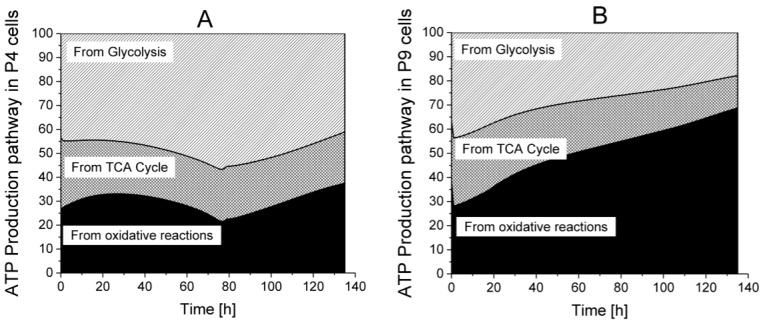
Distribution of ATP turnover origin between glycolysis, TCA cycle and oxidative phosphorylation. (**A**) P4 cell culture; (**B**) P9 cell culture. 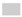 ATP production from glycolysis, 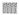 ATP production from TCA cycle, 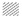 ATP production from respiration.

Among the major roles of the TCA cycle, the recycling of nucleotide shuttles that are fuelling ATP regeneration in the oxidative phosphorylation metabolism is a priority. Model simulations thus allowed questioning cell metabolism in order to determine the specific contributions of glycolysis, TCA cycle, and respiration on ATP turnover rate (Figure 12). In glycolysis, PGK and PK fluxes contribute to ATP recycling, whereas in the TCA cycle, succinyl-CoA synthetase (SCOAS) and SDH are directly contributing in addition to the other reactions feeding proton shuttles to the oxidative phosphorylation reactions leading to massive ATP regeneration. In P4 cells, glycolysis accounts for more than 45% of total ATP turnover while in P9 cells, ATP is mainly recycled via the oxidative phosphorylation metabolism. This difference in ATP turnover rate as well as its metabolic location may both represent efficient biomarkers of cell phenotypic loss with passages: early passage cells exhibit a glycolytic metabolism with a high lactate-to-glucose ratio, whereas late passage senescent cells show increasing respiratory metabolism. These results are in agreement with other works, suggesting that glycolysis is the main energetic support for healthy stem cells [40,41].

**Figure 13:**
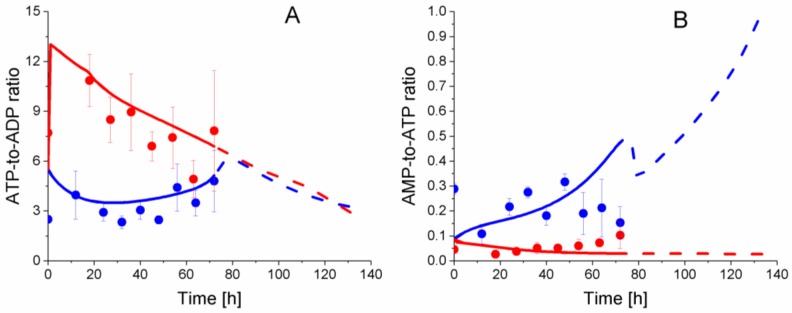
Effect of cell passage on energetic nucleotides ratio. (**A**) ATP-to-ADP ratio; (**B**) AMP-to-ATP ratio. Same conditions and symbols as in Figure 3 are applied.

In this work, energetic and redox nucleotides were quantified and simulated by the model, as concentrations (Supplementary Materials, Figure S1), as well as energetic ratios (Figure 13). Interestingly, comparing nucleotide concentration showed no clear differences between P4 and P9 cells (Figure S1), but the analysis of nucleotide ratio reveals clear distinct behaviour with cell age. In P9 cells, ATP-to-ADP ratio regularly decreases from 13.0 to 5.1, while this energetic ratio stays constant and at a lower level in P4 cells; between 2.3 ± 0.6 to 4.8 ± 3.2, as observed experimentally and simulated by the model. The AMP-to-ATP ratio in P9 cells, however, shows a constant slight increase from 2.6 ± 0.5 × 10^−2^ to 10.2 ± 9.2 × 10^−2^, but to a lower level than in P4 cells in which this ratio evolves with no clear trend between 2.9 ± 0.3 × 10^−1^ and 1.5 ± 1.1 × 10^−1^. Interestingly, the simulation of the ATP-to-ADP ratio closely reflects experimental data, while the model simulation trends slightly differ from experimental values for the AMP-to-ATP ratio. The ATP and ADP concentrations agree with previous results in CHO cell lines [28,30,31], and lead to similar ATP-to-ADP ratio levels. However, the AMP concentration is five times higher in WJMSCs than CHOs with a value of ~5 × 10^−7^ mmol·10^−6^ cells. Taking the higher ATP-to-ADP ratio observed (and simulated) in P9 cells, results that are consistent with a respiratory metabolism, the expected low AMP-to-ATP ratio is observed (and simulated). Interestingly, both experimental ATP-to-ADP and AMP-to-ATP ratios tend to reach similar values at the end of the cultures, which coincided with P4 cell confluency and growth rate reduction (see above). Hardie et al. proposed a high AMP-to-ATP ratio as a marker of oxidative stress [42]. Moreover, they demonstrated that an oxidative stress could activate the AMP-activated protein kinase (AMPK), leading to the inhibition of ATP-consuming pathways such as glucose uptake, reduction of cell growth, and the activation of efficient ATP-producing pathways [43,44]. Thus, our results on the energetic nucleotides suggest the WJMSCs immunomodulatory phenotype can support a higher oxidative stress level, a hypothesis also supported by a glycolytic metabolism. However, the change of phenotype impedes P9 cells to support such oxidative stress level, which cause a switch from a glycolytic to a respiratory metabolism, as observed in this work.

### 2.7. P4 Cells Show a Higher Urea Cycle Activity

**Figure 14:**
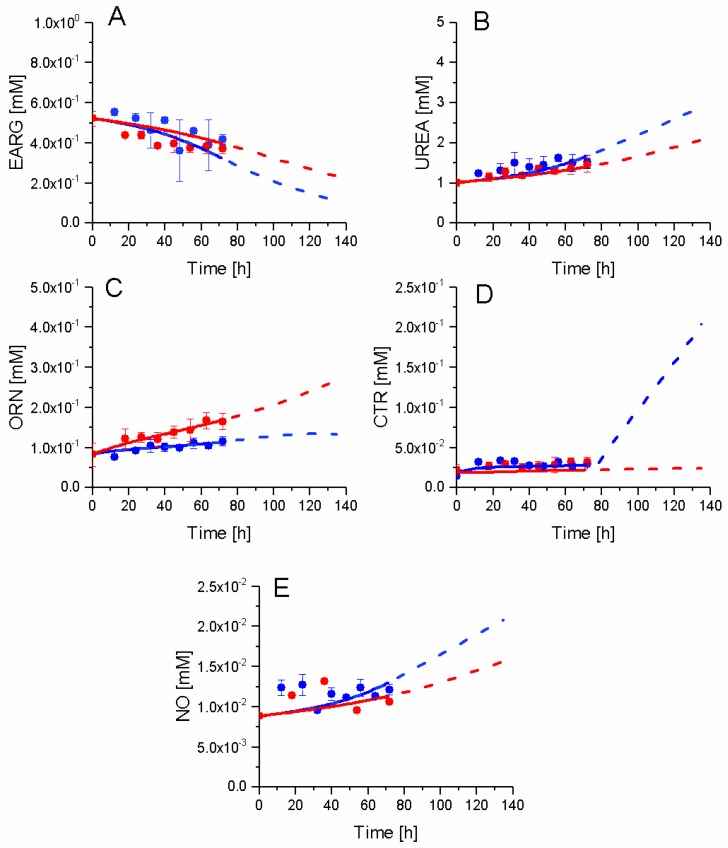
Effect of cell passage on urea cycle intermediates. (**A**) Extracellular arginine concentration; (**B**) Extracellular urea concentration; (**C**) Ornithine concentration; (**D**) Citrulline concentration; and, (**E**) Nitric oxide concentration. Same conditions and symbols as in Figure 3 are applied.

The urea cycle activity is known to be modulated with the immunosuppressive activity [9,45]. In this work, although MLR data showed no significant differences in the immunosuppressive activity between P4 and P9 cells, in the sections above, our results have demonstrated distinct metabolic behaviours (Figure 14). Indeed, P4 cells show a tendency towards expressing some immunosuppressive traits at higher levels than P9 cells when comparing glycolysis, TCA, and energetic behaviours. Experimental data of extracellular arginine (EARG), urea, and nitric oxide (NO), as well as intracellular citrulline (CTR), show similar trends for both P9 and P4 conditions, although the concentration of ornithine (ORN) in P9 cells is increased as compared to P4 cells. The differences in behaviour were simulated by the model for ORN, while the model simulates slight differences of behaviour for the other metabolites. Interestingly, simulations show a higher consumption of EARG and a higher production of NO and UREA in P4 cells. A high EARG consumption and NO production are markers of immunosuppressive activity [8,46]; it thus seems that the passage number effects overlaps with changes in the immunosuppressive phenotype. The rapid increase of citrulline from 72 h may be a consequence of the simplicity of description of this urea cycle sub-network, as well as growth cessation in P4 cells, a phenomenon reducing the cell consumption of intracellular metabolites. In the case of NO, we have previously shown such NO increase in bone marrow-derived MSCs in culture [34] is even more pronounced when the immunosuppressive function is strong. The slow increase observed and reported here may be due to the weak immunosuppressive trait of P4 and P9 cells.

**Figure 15:**
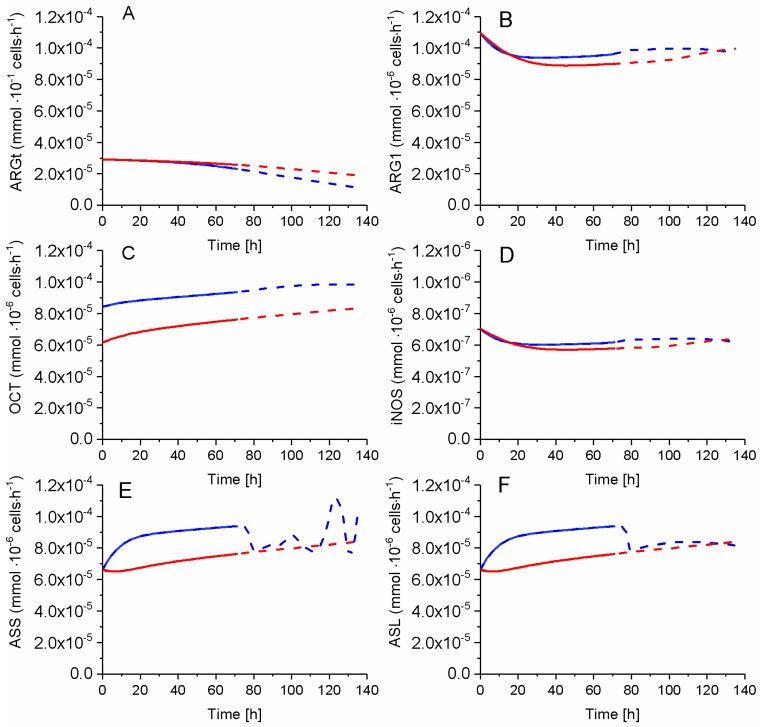
Urea cycle fluxes. (**A**) Arginine transferase flux; (**B**) Arginase-1 flux; (**C**) Ornithine carbamoyltransferase flux; (**D**) Inducible nitric oxide synthetas flux; (**E**) Argininosuccinate synthase flux; and, (**F**) Argininosuccinate lyase flux. Same conditions and symbols as in Figure 3 are applied.

While ARGt activity in P9 cells remains higher and diverges from that in P4 cells, all other enzymes of urea cycle show a higher activity in the P4 cells when compared to P9 cells (Figure 15). Sensitive parameter $vmax_{OCT}$ decreased 0.7x for P9 cells, but P4 OCT activity slightly and continuously increased with time while staying higher than in P9 cells. ARG1 and iNOS, on the other hand, exhibit a similar time-profile in both cells. ASS and ASL activities are higher in P4 cells before 80 h (i.e., at growth cessation), before reaching similar values than P9 cells. Thus, the urea cycle is more active in P4 as compared to P9 cells, agreeing with higher consumption rate of EARG and production rate of NO, which are two known biomarkers of a cell immunosuppressive activity [8,9,39]. Thus, in agreement with above-mentioned results, P4 cells exhibit higher levels of known biomarkers of an immunosuppressive phenotype when compared to P9 cells with, however, clear evidence that the cell passage effect overlaps with that of immunosuppressive phenotype, such as a more pronounced NO concentration increase is observed in highly immunosuppressive MDC cells [8,9,39].

### 2.8. P4 Cells Consume Less Tryptophan

**Figure 16:**
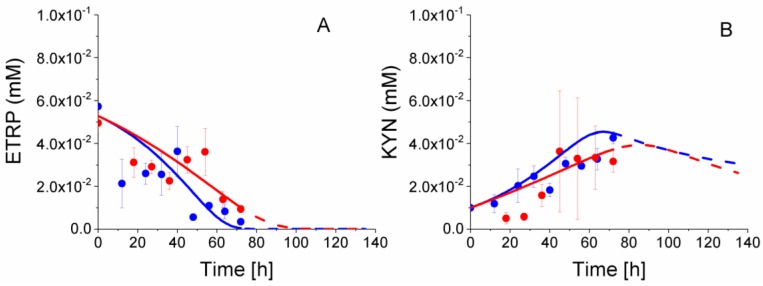
Effect of cell passage on tryptophan consumption. (**A**) Extracellular tryptophan concentration; (**B**) Extracellular kynurenine concentration. Same conditions and symbols as in Figure 3 are applied.

Tryptophan metabolism is also well known as a key biomarker of an immunosuppressive phenotype [6,47]. Indeed, in agreement with our MLR data, experimental concentration data show a faster ETRP consumption in P4 cells (Figure 16). P4 ETRP concentration continuously decreases, with model simulations suggesting this essential amino acid being a potential limiting nutrient of cell growth (from model simulations). At the opposite, KYN concentration increased during P4 and P9 cells growth, for decreasing from ETRP depletion (estimated from model simulations).

**Figure 17:**
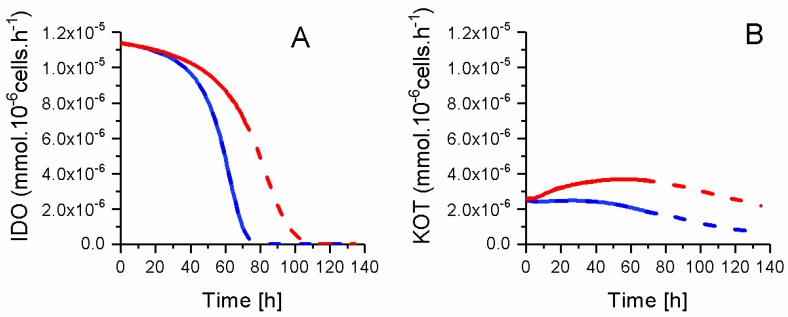
ETRP metabolism fluxes. (**A**) Indoleamine 2,3-dioxygenase flux; (**B**) Kynureninase flux. Same conditions and symbols as in Figure 3 are applied.

Interestingly, and as previously shown for other metabolic sub-pathways, model simulations allow for obtaining a clearer view on tryptophan metabolism. The model simulates a higher tryptophan specific consumption rate in P9 cells as compared to P4 cells (Figure 17), although one can think the opposite looking at TRYP concentrations (Figure 16). P4 and P9 IDO fluxes both start at a similar value then decreases asymptotically to a zero value ~10 h before respective growth cessation. In the case of kynureninase (KOT) flux, both P4 and P9 cells also start at a similar value, and then increases in P9 cells until 60 h then decreases. KOT flux stayed at a constant value in P4 cells until ~40 h, then decreases with a similar trend as for P9 cells. For instance, and similarly to glucose consumption, P4 cells show a lower IDO activity but ETRP is consumed at a faster rate than for P9 cells due to a higher cell growth rate for P4 cells. This faster tryptophan depletion suggests that P4 cells behave similarly to macrophages that inhibit the T-cell response against maternal foetus [5]. This faster tryptophan depletion in P4 cells, that is a phenomenon representing a known biomarker of immunosuppressive phenotype, may seem contradictory with the higher tryptophan specific uptake rate in P9 cells. However, the immunosuppressive phenotype does not rely on an uptake rate, rather than on the maintenance of a low extracellular concentration in tryptophan. Therefore, in agreement with all data above, P4 WJMSCs show characteristics that are specific to immunosuppressive cells when compared to late P9 cells. However, this hypothesis has been showed to be clearly biased by the phenotype senescence phenomenon induced with cell passages and that is overlapping with the immunosuppressive phenotype.

## 3. Materials and Methods

### 3.1. Wharton’s Jelly Mesenchymal Stem Cells Culture

WJMSCs were first isolated from human umbilical cords at the Ottawa General Hospital leading to a 1st passage (P1) and were transferred to the Ottawa Hospital Research Institute. During the entire process, the cells were inoculated at a cell density of 5000 cells cm^−2^ and cultivated in 75 cm^2^ T-flasks and 150 cm^2^ T-flasks (Life Science, Corning, Tewksbury, MA, USA) with 10 mL and 20 mL of α-MEM (Life Technologies, Burlington, ON, Canada) supplemented with 20% of fetal bovine serum (Life Technologies, Burlington, ON, Canada), 1% of Glutamax (Life Technologies, Burlington, ON, Canada) and 1% of Antibiotic-Antimycotic (Life Technologies, Burlington, ON, Canada), and cultured in a 5% CO_2_, 5% O_2_, and 37 °C humidified incubator. Cells were first amplified at the Ottawa Hospital Research Institute in order to furnish enough cells for further sets of experiments, leading to a 2nd passage (P2). A part of these P2 cells were transferred to Polytechnique on dry ice, whereas the other part was cultivated till 7 passage (P7) before sending. P2 (Second passage) and P7 (seventh passage) WJMSCs were thawed at Polytechnique and cultivated during 12h in the medium previously described to avoid thawing effects. After these 12 h, 15 ng mL^−1^ of TNFα, and 10 ng mL^−1^ of IFNγ were added to the culture medium and cells were cultivated 48 more hours in order to induce cells immunosuppressive properties, leading to P3 and P8 cells. After this 60 h-culture, the cells were passed in order to start the extraction experiment, leading to P4 and P9 cells. For early passage cells, the number of passage (4) was the smallest number possible in front of all the different constraints, whereas for late passage cells, a choice was made to use P9 cells. For each analysis, cells were detached using 50 μL cm^−2^ of TryplE (Life Technologies, Burlington, ON, Canada), rinsed twice with PBS, centrifuged for 5 min at 300 g, and counted with a hematocytometer (Sigma-Aldrich, Oakville, ON, Canada). Every 48 h, immunosuppressive properties of cells were maintained by adding 15 ng mL^−1^ of TNFα and 10 ng mL^−1^ of IFNγ in the culture medium. The protocol was the same for late and early cell cultures, however, the sample volume was adapted to the cultureF cell concentration. Because each sample has to contain at least 3 × 10^6^ cells for adequate metabolite quantification, P4 passage cells were sampled at 0, 12, 24, 32, 40, 48, 56, 64, and 72 h, whereas P9 passage cells were sampled at 0, 18, 27, 36, 45, 54, 63, 72 h in order to obtain enough cells for analysis.

### 3.2. Mixed Lymphocyte Reaction (Mlr) Method

CD3^+^ T lymphocytes were isolated from umbilical cord blood samples (Canadian Blood Services, Montreal, QC, Canada) using a positive selection kit (StemCell Technologies, Vancouver, BC, Canada) and stained with 1 µM CFSE (Molecular Probes, Thermo Fisher, Burlington, ON, Canada) for 5 min. Cells were washed twice with PBS (Invitrogen, Thermo Fisher, Burlington, ON, Canada), resuspended in OpTmizer media (Invitrogen), and 4 × 10^4^ cells per well were seeded in triplicate in a 96 wells plate (Life Science, Corning, Tewksbury, MA, USA) and stimulated with CD3/28 Dynabeads (Invitrogen). MSCs were grown as described above in proinflammatory conditions induced with TNFα and IFNγ, 48 h prior to harvesting. MSCs were resuspended in OpTimizer media and 4 × 10^3^ cells were added to the stimulated T cells. Co-cultures were placed at 37 °C in a humidified incubator, at 5% CO_2_ and 5% O_2_, for five days. To analyze T lymphocyte proliferation, cells were stained with anti-CD45 antibodies (clone 2D1) conjugated to AlexaFluor700 (eBioscience, Thermo Fisher, Burlington, ON, Canada) and 10 ng µL^−1^ propidium iodide (Sigma) and analyzed using a BDFortessa flow cytometer. Stimulated CFSE fluorescence of CD3^+^CD45^+^ cells, in the absence or presence of MSCs, were compared to determine T cell proliferation.

### 3.3. Intracellular Metabolites Extraction

The extraction protocol was adapted from Hammami et al. [39] based on a method developed by Kimball et al. [48]. Briefly, for each sample, the cells were extracted with 400 μL of 80% cold methanol in the presence of 0.2 g of sand (Sigma-Aldrich). After 30 min in dry ice, the mixture was vortexed at high velocity and then sonicated in ice and water for 5 min. Samples were then centrifuged for 7 min at 10,000× *g* and 4 °C to collect supernatants. The pellets were extracted a second and third time as described above with 200 μL of 50% cold methanol and 200 μL of ice-cold water, respectively. Supernatants were mixed and stored at −80 °C prior to analysis. Extracts were filtered through 0.2 μm PTFE filters (Millipore, ON, Canada) before analysis.

### 3.4. Nucleotide Concentration

Nucleotides in WJMSCs extracts were analysed following the method developed in Hammami et al. [39]. A minimum of 3 million cells was needed at each extraction in order for metabolite concentrations to be higher than the minimum threshold of this method. Briefly, the extracts were analyzed using a 1290 UPLC system coupled to a 6460 triple quadruple mass spectrometer (both from Agilent Technologies, St. Laurent, QC, Canada). Nucleotides were separated by Symmetry C18 column (150 × 2.1 mm, 3.5 μm) (Waters) and a Security C18 guard-column (Water, 10 × 2.1 mm, 3.5 μm) by the ion-pair method. DMHA (*N*,*N*-dimethylhexylanine, Sigma) was used as an ion-pair reagent to improve the signal-to-noise ratio with positive ionization mode. The mobile phase consisted of Buffer A: 10 mM ammonium acetate, 15 mM DMHA at pH 7.0, and buffer B: 40/60% (*v*/*v*) acetonitrile. Mobile phase flow rate was set at 0.3 m/min with the following gradient: 0–10 min at 10% B, 10–20 min at linear gradient from 10 to 30% B, 20–21 min at linear gradient from 30 to 60% B, 21–26 min at 60% B, 26–27 min with a linear gradient from 60 to 10% B and 27–35 min at 10% B.

### 3.5. Organic Acid Concentration

As well as the previous described method, a minimum of 3 million cells were needed at each extraction in order for organic acids concentrations to be higher than the minimum threshold of this device. Organic acids concentrations were assessed using the above-mentioned UPLC-MS/MS system using a Hypercarb column (100 × 2.1 mm, 5 um) and a Hypercarb precolumn (2.1 × 10 mm, 5 um) (Thermo Fisher, Burlington, ON, Canada). The mobile phase consisted of Buffer A, 20 mM ammonium acetate at pH 7.5, and Buffer B, 10% (*v*/*v*) methanol in water. The flow rate was set at 0.3 mL/min, with the following gradient: 0–5 min at 10% A, 5–10 min at linear gradient from 10% to 20% A, 10–20 min at linear gradient from 20% to 100% A, 20–30 min at 100% A, 30–32 min at linear gradient from 100% to 10% A and 32–40 min at 10% A. Quantification of metabolites (nucleotides and organic acids) was performed by integrating peak areas and using calibration curves. Cell specific concentrations in metabolites were calculated by normalizing the quantity of metabolites in cell extracts to the number of extracted cells.

### 3.6. Extracellular Amino Acid Concentration

Extracellular samples were collected and diluted 1:10 in milli-Q water, then 1:10 with 50% methanol and finally 1:1 with amino acids ISTD. Samples were filtered through 0.2 μm PTFE filters (Millipore, ON, Canada) before analysis.

The analysis of amino acids concentration was performed on an Agilent 1290 UPLC system (Agilent technologies, Montreal, QC, Canada) coupled to an Agilent 6460 triple quadruple mass spectrometer (Agilent technologies, Montreal, QC, Canada), using previously described methods [49,50]. The underivatized amino acids were separated by a ZIC-Hilic column (3.5 mm, 200 A, PEEK) (Merck SeQuant, Peterborough, Canada) and a ZIC-Hilic guard column (5 mm, 200 A, PEEK) (Merck SeQuant, Peterborough, ON, Canada) at a column temperature of 35 °C and injection volume of 2 µL. The mobile phase buffer contained 200 mM HCOONH_4_ (Sigma, cat. # 74314) at pH 4. The mobile phase A contains 10% of the mobile phase buffer in water, and the mobile phase B contains 10% of the mobile phase buffer in acetonitrile (ACN) (Sigma, cat. # A3396). The mobile phase B was linearly decreased from 90% to 35% in 19 min, and then was increased to 90% in one minute and held at 90% for 15 min at a flow rate of 0.1 mL min^−1^. The Agilent 6460 triple quadruple mass spectrometer (Agilent technologies), equipped with a Jet sheet stream electrospray ion source (Agilent technologies), was used for the analysis of amino acids in positive ion mode. The other parameters: gas temperature of 350 °C, gas flow rate of 9 L min^−1^, nebulizer pressure of 45 PSI, sheath gas temperature of 350 °C, sheath gas flow rate of 10 L min^−1^, and capillary votage of 3 kV. An internal standard solution that contains 2 µM homoarginine (Fisher cat.# AC169090010), 2 µM homophenylalanine (Sigma cat.# 294357), and 2 µM methionine-d3 (CDN isotope D1292) was used as internal standard for quantification. The MRM transition and retention time of each amino acid is listed in Table S4 (Supplementary Materials). It should be noted that commercial standards of every medium compound and metabolites quantified were also used to establish calibration curves along with each series of analysis. Finally, extraction efficiency and compounds stability were determined using internal standards. Only amino acids showing significant concentration variation are shown.

### 3.7. Extracellular Nitric-Oxide Analysis

Nitric oxide (NO) concentration in supernatant was assessed using a Nitrate/Nitrite Colorimetric Assay Kit (Cedarlane, Missisauga, ON, Canada). The absorbance was measured at 540 nm with a Victor 3V multilabel reader (PerkinElmer, Waltham, MA, United States).

### 3.8. Metabolic Model Structure

**Figure 18:**
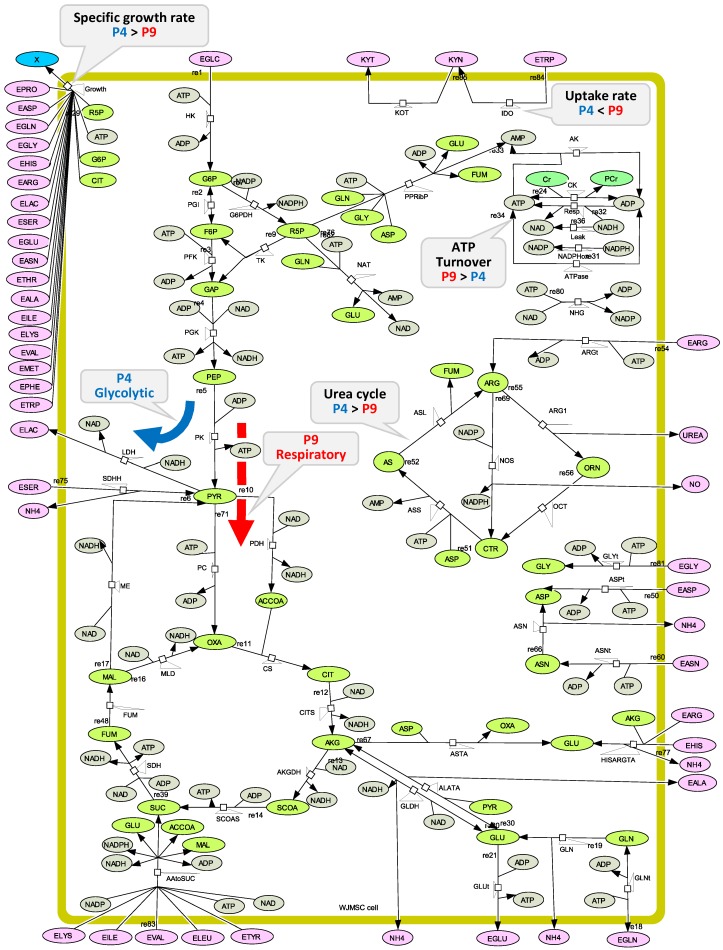
The metabolic network. Draw with the CellDesigner^TM^ modeling tool for biochemical networks.

The global modelling approach previously validated for Chinese Hamster Ovary (CHO) cell lines [31] has been adapted to describe Wharton’s Jelly mesenchymal stem/stromal cells. The metabolic network of reactions (Figure 18) composed of 49 enzymatic reactions (Table S1, Supplementary Materials) includes the biochemical pathways of the central carbon metabolism as well as pathways that are specifically involved in the immunosuppression phenomenon. The model thus describes the glycolysis, the TCA cycle and the pentose phosphate pathway (PPP), as well as the energetic metabolism. The latter includes the oxidative phosphorylation and other production and consumption mechanisms, such as when energetic nucleotides are involved as co-factors as well as for fuelling anabolic reactions. Amino acid metabolism also considered: glutaminolysis with reactions involving aspartate and alanine transaminase (*V_ASTA_* and *V_AlaTA_*), serine conversion to pyruvate (*V_SDHH_*), as well as amino acid conversion to alpha-ketoglutarate and succinate (respectively in *V_AAtoSUC_* and *V_HISARGTA_*). The urea cycle is considered, as well as the reaction involving indoleamine 2,3-dioxygenase (IDO) to account for well-known immunosuppressive-related metabolic pathways. In total, six metabolic reactions were considered to describe the urea cycle: the inducible nitric oxide synthetase (iNOS), aginase-1 (ARG1), aginase Tranferase (ARGt), argininosuccinate lyase (ASL), argininosuccinate synthase (ASS), and ornithine carbamoyltransferase (OCT). This set of reactions was taken from Morris et al. [51]. The TCA cycle is described with reactions leading to citrate (CIT), alpha-ketoglutarate (AKG), succinyl-CoA (SCOA), succinate (SUC), fumarate (FUM), malic acid (MAL), and oxaloacetate (OXA). Moreover, metabolic pathways involving amino acids are detailed with reactions catalyzed by the enzymes asparaginase (ASN), asparagine transporter (ASNt), aspartate transporter (ASPt), as well as glutamine transporter (GLNt), glutamate transporter (GLUt), glycine transporter (GLYt), and glutaminase (GLNase). Only amino acids exhibiting a significant change in time have been considered in the model. Moreover, the reaction involving creatine kinase (CK) is described. Finally, global lumped reactions for AMP synthesis from phosphoribosyl pyrophosphate (PPRibP) enzyme as well as NAD and NADP synthesis, respectively, from simplified pathways involving NAD(P)+ nucleosidase (NAT) and NAD-Kinase (NHG) are also described. Nucleotide synthesis is required since cell division implies dilution of their intracellular content. The specific growth rate kinetics and stoichiometry were taken from Robitaille et al. who described the CHO bio-system [31]. Briefly, cell composition was taken from Sheik et al. [52] who developed a model for mammalian cells, and cell precursors of glycogen, lipids, and nucleotides synthesis were assumed to be issued from glucose-6-phosphate (G6P), citrate (CIT), and ribulose-5-phosphate (R5P), respectively. The cell proteins were considered to have an average weight of 107.5 g·mol^−1^ and the cell biomass a molar weight of 3.15 × 10^−4^ gDW·10^−6^ cells. Finally, enzymatic reactions were kinetically translated, as described in the Appendix A, leading to the set up of flux equations (Table S5, Supplementary Materials), as well as metabolite concentrations differential equations (Table S6, Supplementary Materials).

## 4. Conclusions

In addition to providing a wide new set of metabolomic data comparing the immunosuppressive phenotype of late passage WJMSC cells (P9) to earlier passage cells (P4), the present study has led to the development of a dynamic metabolic model that is specifically adapted to describe WJMSCs. The model metabolic network includes the central carbon metabolism, with the glycolysis, PPP, and TCA cycle, the energetic metabolism as well as pathways that are known to be differentially involved in immunosuppressive cells. Complementing the analysis of metabolomics data, model simulations enabled highlighting that P4 cells still exhibit an immunosuppressive-related metabolism. A series of coherent biomarkers were thus identified pointing out that immunosuppressive cells may favour a glycolytic metabolism, gradually switching to an oxidative phosphorylation metabolism while progressing with cell passages, from the forth (P4) to the ninth (P9) in the present study. The time-evolution of the AMP-to-ATP ratio also suggests that P9 cells have a lower tolerance to oxidative stress conditions. Moreover, even if P4 cells are not significantly immunosuppressive compared to P9 cells, we have demonstrated that P4 cells still express some biomarkers of an immunosuppressive phenotype that is normally seen at early passages WJMSCs (<P4). However, we have also clearly shown that the accumulation of cell passages leads to the overlap of immunosuppressive and senescence phenotypes, a result justifying the importance to better understand WJMSC metabolism specifically associated to the senescence phenotype. Indeed, more work is required with early passage cells, such as P2 and P3, to enable a clear characterization of WJMSC immunosuppressive metabolism. Finally, further work on the description of flux regulation [53,54] is expected to allow the dynamic metabolic model to further tease apart cell metabolism associated with immunomodulatory function versus cell growth, and would lead to the discovery of specific immunosuppressive metabolic biomarkers. The dynamic metabolic model developed in this work can thus become a useful tool to further describe the identity of immunosuppressive cells, as well as to improve culture conditions, such as medium composition in essential nutrients and amino acids.

## Figures and Tables

**Table 1:** Values of sensitive parameters according to cell passage.

Parameters	Units	P4 Cells	P4 and P9 Combined	P9 Cells	P9 vs. P4
$vmax_{AAtoSUC}$	mmol·10^−6^ cells·h^−1^	6.20 × 10^−4^	6.20 × 10^−4^	1.34 × 10^−2^	22x
$vmax_{AK}$	mmol·10^−6^ cells·h^−1^	1.87 × 10^−4^	1.92 × 10^−4^	1.94 × 10^−4^	idem
$vmax_{AKGDH}$	mmol·10^−6^ cells·h^−1^	1.53 × 10^−3^	1.53 × 10^−3^	1.53 × 10^−3^	idem
$vmax_{CITS}$	mmol·10^−6^ cells·h^−1^	9.42 × 10^−4^	1.08 × 10^−3^	1.11 × 10^−3^	1.2x
$vmax_{CS}$	mmol·10^−6^ cells·h^−1^	9.45 × 10^−4^	1.08 × 10^−3^	1.10 × 10^−3^	1.2x
$vmax_{growth}$	h^−1^	8.57 × 10^−2^	6.97 × 10^−2^	4.26 × 10^−2^	0.5x
$vmax_{HK}$	mmol·10^−6^ cells·h^−1^	1.58 × 10^−3^	1.00 × 10^−3^	7.67 × 10^−4^	0.5x
$vmax_{LDH}$	mmol·10^−6^ cells·h^−1^	7.91 × 10^−5^	8.80 × 10^−5^	8.88 × 10^−5^	idem
$vmax_{NADPHox}$	mmol·10^−6^ cells·h^−1^	4.19 × 10^−5^	5.33 × 10^−5^	7.81 × 10^−5^	1.9x
$vmax_{OCT}$	mmol·10^−6^ cells·h^−1^	1.35 × 10^−4^	1.42 × 10^−4^	9.89 × 10^−5^	0.7x
$vmax_{PDH}$	mmol·10^−6^ cells·h^−1^	1.63 × 10-^03^	1.17 × 10^−3^	1.08 × 10^−3^	0.7x
$vmax_{resp}$	mmol·10^−6^ cells·h^−1^	7.09 × 10^−4^	7.20 × 10^−4^	1.20 × 10^−3^	1.7x
$vmax_{SDH}$	mmol·10^−6^ cells·h^−1^	1.39 × 10^−3^	1.39 × 10^−3^	1.39 × 10^−3^	idem
$vmax_{ASTA}$	mmol·10^−6^ cells·h^−1^	3.16 × 10^−4^	3.16 × 10^−4^	3.16 × 10^−4^	idem
$vmax_{GLN}$	mmol·10^−6^ cells·h^−1^	1.79 × 10^−1^	1.79 × 10^−1^	1.79 × 10^−1^	idem
$Km_{AAtoSUC_{AKG}}$	mM	1.83 × 10^−7^	1.00 × 10^−7^	5.21 × 10^−7^	2.8x
$Km_{AAtoSUC_{EILE}}$	mM	3.00 × 10^−1^	3.00 × 10^−1^	3.35 × 10^−1^	idem
$Km_{AAtoSUC_{ELEU}}$	mM	3.00 × 10^−1^	3.00 × 10^−1^	9.70 × 10^−1^	3.2x
$Km_{AAtoSUC_{ELYS}}$	mM	3.00 × 10^−1^	3.00 × 10^−1^	8.47 × 10^−1^	2.8x
$Km_{AAtoSUC_{ETYR}}$	mM	1.50 × 10^−1^	1.50 × 10^−1^	2.31 × 10^−1^	1.5x
$Km_{AAtoSUC_{EVAL}}$	mM	5.00 × 10^−1^	5.00 × 10^−1^	2.49 × 10^−1^	0.5x
$Km_{CS_{ACCOA}}$	mmol·10^−6^ cells	1.00 × 10^−8^	1.00 × 10^−8^	1.00 × 10^−8^	idem
$Km_{HK_{EGLC}}$	mM	4.95	5.00	6.06	1.2x
$alpha_{HK_{AMP_{ATP}}}$	/	1.05	1.10	1.28	1.2x
$beta_{HK_{AMP_{ATP}}}$	/	6.27 × 10^−1^	1.05	1.69	2.7x
$Km_{LDH_{PYR}}$	mmol·10^−6^ cells	1.12 × 10^−7^	1.08 × 10^−7^	1.04 × 10^−7^	idem
$alpha_{LDH_{AMP_{ATP}}}$	/	4.57 × 10^−1^	4.65 × 10^−1^	4.68 × 10^−1^	idem
$beta_{LDH_{AMP_{ATP}}}$	/	1.38 × 10^1^	1.20 × 10^1^	1.19 × 10^1^	idem
$Km_{PDH_{PYR}}$	mmol·10^−6^ cells	4.06 × 10^−7^	2.00 × 10^−7^	2.00 × 10^−7^	0.5x
$Km_{SDH_{SUC}}$	mmol·10^−6^ cells	3.00 × 10^−7^	3.00 × 10^−7^	3.00 × 10^−7^	idem
$vgrowth_{ATP}$	mmol·10^−6^ cells	1.19 × 10^−2^	1.19 × 10^−2^	1.19 × 10^−2^	idem
$vgrowth_{ADP}$	mmol·10^−6^ cells	1.19 × 10^−2^	1.19 × 10^−2^	1.19 × 10^−2^	idem

**Table 2:** Experimental and model simulated specific growth rates.

Cells	Experimental Data (h^−1^)	Model Estimations (h^−1^)
P4	[2.4 ± 0.3] × 10^−2^	[2.1–2.5] × 10^−2^
P9	[1.5 ± 0.3] × 10^−2^	[1.15–1.24] × 10^−2^

## Supplementary Materials

The following are available online at http://www.mdpi.com/2218-1989/8/1/18/s1, Model SBML file, Table S1: Reaction equations, Table S2: Successive steps for model calibration, Table S3: Parameter values for average simulation, Table S4: MRM transition and retention time of each amino acid, Table S5: Flux equations, Table S6: Metabolite concentrations differential equations, Figure S1: Experimental data and model simulation of cofactors and nucleotides concentration.

## Appendix A. Kinetic Description of the Metabolic Fluxes

Every intracellular metabolite concentration is described by a material balance (Equation (A1)):

(A1) $$\frac{\mathrm{dM}}{\mathrm{dt}}=S\cdot \nu -\;\mu \cdot M-\mu \cdot \varepsilon$$

where S is the stoichiometric matrix of the network fluxes, ν is the vector of the reaction flux (mmol·gDW^−1^·h^−1^), μ is the specific growth rate (h^−1^), and M is the vector of the 59 metabolite concentrations. The cell dilution term (μ·M) describes the dilution of intracellular metabolites from cell division of a mother cell into two daughter cells, each with similar final volume and composition. Moreover, the consumption of intra and extracellular metabolites into cell material for growth is also described with (μ·ε) where ε is the stoichiometric coefficient of the “*i*” metabolite integrated into biomass (i.e., no more available to reactions).

The biomass evolution is described as follows (Equation (A2)):

(A2) $$\frac{dX}{dt}=\mu \cdot X$$

where X is cell concentration (10^6^ cells mL^−1^), and “µ” is the specific growth rate (h^−1^).

Every biochemical reaction (i.e., $\nu_{i}$ flux rate) is mathematically described according to the Michaelis-Menten kinetic representation, as previously described and validated for eukaryotic cells [28,30,31]. All metabolites involved in the reaction network, either as substrate or co-factor, are considered with Michaelis-Menten constants specific to a substrate and a reaction. When the substrate (or the co-factor) is an energetic nucleotide, such as NADH, NADPH, NAD, NADP, ATP, ADP, AMP, ratios are used to improve model interpretation of kinetics fluxes as previously demonstrated for various cell biosystems [28,30,31,55] (Equation (A3)). The cell specific growth rate (μ) has been approximated applying Monod kinetics with multiple intracellular substrates such as the major precursors of cell building blocks, as successfully applied in previous works [28,30,31,56] (Equation (A4)).

(A3) $$\nu_{E_{j}}=\;\nu_{E_{j},max}\cdot \underset{i}{\prod }\frac{M_{i}}{M_{i}+K_{m_{E_{j}M_{i}}}}$$

(A4) $$\mu =\nu_{growth}=\nu_{growth,max}\cdot \underset{j}{\prod }\frac{M_{j}}{{K_{m}}_{growth,M_{j}}+M_{j}}$$

where $\nu_{E_{i}}$ is the flux rate (mmol 10^−6^ cells h^−1^) of the enzymatic reaction catalysed by the enzyme $E_{i}$, $\nu_{E_{i},max}$ is the maximum reaction rate of the flux catalyzed by the enzyme $E_{i}$ (mmol 10^−6^ cells h^−1^), $M_{i}$ are each substrate and co-factors of *M* considered in flux kinetics, μ is the cell specific growth rate (h^−1^), $\nu_{growth,max}$ is the cell maximum specific growth rate (h^−1^), $M_{j}$ are each substrate and co-factors of *M* considered in cell growth kinetics, and $K_{m_{E_{j}M_{i}}}$ and ${K_{m}}_{growth,M_{j}}$ are the Michaelis-Menten constants respectively for the $M_{j}$ involved in the cell specific growth rate and in the cell biomass synthesis, respectively.

As proposed in Nolan and Lee [32], exchanges of metabolites between intracellular and extracellular volumes were not considered to be controlled by membrane protein transporters but by intracellular enzymes. This model simplification is also supported considering that the extracellular concentration of the metabolites is higher than their associated membrane transporter half-saturation Michaelis-Menten constant [57]. Thus, the uptake or secretion of extracellular metabolites is controlled by several intracellular reactions rather than their transporter.

As in previous works [28,30,31], the main regulation mechanisms of activation or inhibition were considered in the flux kinetics. Indeed, on the one hand, hexokinase ($v_{HK}$), phosphoglucose isomerase ($v_{PGI}$) and reverse lactate dehydrogenase ($vr_{LDH}$) are respectively inhibited by glucose-6-phosphate (G6P), phosphoenolpyruvate (PEP) and pyruvate (PYR). On the other hand, phosphofructokinase ($v_{PFK}$), lactate dehydrogenase ($v_{LDH}$) and hexokinase ($v_{HK}$) are activated by the AMP-to-ATP ratio, and finally, pyruvate kinase ($v_{PK}$) is activated by fructose-6-phosphate (F6P). The reactions involving an inhibition or activation mechanism were respectively described according to the mathematical formulation of a non-competitive inhibition (Equation (A5)) and a non-essential activation (Equation (A6)) impacting the Michaelis-Menten mechanism of the main substrate, respectively, as described in Robitaille et al. [31].

(A5) $$V_{E_{j}}=\;V_{E_{j},max}\cdot \;\underset{i}{\prod }\frac{M_{i}}{M_{i}+K_{m_{E_{j}M_{i}}\;}}\;\cdot \left(\frac{I+K_{i}{}_{E_{j}I}}{I}\right)$$

(A6) $$v_{E_{j}}=\;\nu_{E_{j},max}\cdot \;\underset{i}{\prod }\frac{M_{i}}{M_{i}+K_{m_{E_{j}M_{i}}\;}}\;\cdot \left(\frac{M_{p}\cdot \left(1+\frac{\beta A}{\alpha .K_{a}}\right)}{K_{m_{E_{j}M_{p}}\;}\cdot \left(1+\frac{A}{K_{a}}\right)+M_{p}\cdot \left(1+\frac{A}{\alpha .K_{a}}\right)}\right)$$

with I the inhibitor concentration and ${K_{i}}_{E_{j}I}$ the inhibition constant, A is the activator concentration, $M_{p}$ the main substrate of the activated reaction, and α, β and $K_{a}$ the activation constants.

## Appendix B. Model Structure and Parameters Value Calibration

The determination of parameters value was performed following an approach adapted from the step-by-step method, through model decomposition into sub-networks, developed by Rizzi et al. [58]; starting with values from a previous work with CHO cells [31] as initial estimates. This approach allows minimizing manual manipulations by optimizing parameters value one sub-network at the time. At first, polynomial time-functions describing experimental data sets time-evolution of the various medium components and intracellular metabolites are determined, and used to define associated mass balances in the Ordinary Differential Equations (ODE) system. These mathematical functions, determined using the *polyfit* function of Matlab, only depend on the variable time. The amount of parameters value to be determined is thus significantly lowered. It is important to note that time evolution simulations of the non-measured metabolites were considered adequate when simulated within ranges found in literature, taking initial concentration from literature. Then, as the first step, a single metabolite only involved in one specific reaction is selected, and the polynomial function describing the time-evolution of this metabolite concentration is replaced by the appropriate biokinetic function. The value of the parameters used in this single reaction are then determined minimizing the following objective function (Equation (A7)) which defines model simulation error compared to experimental data sets (solved using the Matlab optimization function lsqnonlin.m):

(A7) $$\min\left(\overset{N}{\underset{n=1}{\sum }}{\left(\frac{X_{n}^{EXP}-X_{n}^{MODEL}\left(p\right)}{\sigma_{n}}\right)}^{2}\right)$$

where p is the vector of the parameters involved in the undetermined reaction, X is the experimental concentration of the metabolite used to find these parameters, N is the number of experimental data points for the metabolite X, $X_{n}^{EXP}$ is the *n*th experimental data of X, $X_{n}^{MODEL}\left(p\right)$ is the model simulated value corresponding to the *n*th experimental value depending on p, and finally $\sigma_{n}$ is the standard deviation of the experimental measurement for each metabolite. Then, this procedure is repeated only to metabolites involved in one reaction, one at the time, keeping with the already determined parameters value but with time-evolution function for the others remaining to be determined. Metabolites simultaneously involved in two reactions were studied following the same procedure but one reaction at the time being kinetically released. In the case of complex cycles and parallel pathways, one metabolite was used to determine two reactions in a single step. The successive steps used to calibrate this model are summarized in Table S2 (Supplementary Materials). In some cases, intracellular metabolite concentration could not be determined experimentally and the initial concentration value was then taken from the value range normally found in literature. Each optimized parameter value was verified for its biologically relevancy, from ranges found in literature.

## Appendix C. Sensitivity Analysis

Once the parameters values were all determined, a sensitivity analysis was performed to identify sensitive parameters, i.e., parameters having a large influence on model simulation. The analysis method was taken from a previous work of Ghorbaniaghdam et al. [30]. The effect of varying parameters value of ±15%, ±25% and ±50% from their optimal value on the global objective function (Equation (A8)) allowed identifying sensitive parameters.

(A8) $$\Delta =\overset{N}{\underset{n=1}{\sum }}\overset{M}{\underset{m=1}{\sum }}{\left(\frac{X_{n,m}^{EXP}-X_{n,m}^{MODEL}\left(P\right)}{X_{n,m}^{EXP}}\right)}^{2}$$

with Δ the model output variation, *N* the number of experimental data points for each metabolite, *M* the number of metabolites, $X_{n,m}^{EXP}$, the *n*th experimental value for the concentration of the metabolite m, and $X_{n,m}^{MODEL}\left(P\right)$ the simulated value corresponding to the *n*th experimental data for the metabolite m calculated with the set of parameter values P. Highly sensitive parameters were those showing a variation of Δ higher than 10% for a small variation of ±15% of their value. However, this sensibility analysis was done with variation higher than 15% in order to validate the sensibility of the sensitive parameters.
