# Supplementary material for: Identifying Biomarkers of Wharton’s Jelly Mesenchymal Stromal Cells Using a Dynamic Metabolic Model: The Cell Passage Effect
Source: Metabolites. 2018 Feb 24;8(1):18. doi: 10.3390/metabo8010018 (PMC5876007; doi:10.3390/metabo8010018)
Supplement: Supplementary file 1 [file metabolites-08-00018-s001.pdf]

metabolites

MDPI

Supplementary Materials: Identifying biomarkers of Wharton's Jelly mesenchymal stem cells using a dynamic metabolic model: The cell passage effect

Benoît Laflaquiere, Gabrielle Leclercq, Chandarong Choey, Jingkui Chen, Sabine Peres, Caryn Ito, Mario Jolicoeur

Table S1. Reaction equations

Reaction number	Reaction name	Reaction
1	AAtoSUC	ELYS + EILE + ELEU + EVAL + ETYR + 7 AKG + ATP + 9 NAD + 2 NADP => 4 GLU + 3 SUC + MAL + 8 ACCOA + ADP + 9 NADH + 2 NADPH
2	AK	ATP + AMP => 2 ADP
3	AKGDH	AKG + NAD => SCOA + NADH
4	ALATA	GLU + PYR = AKG + EALA
5	ARG1	ARG => UREA + ORN
6	ARGt	EARG + ATP => ARG + ADP
7	ASL	AS => FUM + ARG
8	ASN	ASN = ASP + NH4
9	ASNt	EASN + ATP => ASN + ADP
10	ASPt	EASP + ATP => ASP + ADP
11	ASS	CTR + ASP + ATP => AS + AMP
12	ASTA	ASP + AKG = OXA + GLU
13	ATPase	ATP => ADP
14	CITS	CIT + NAD => AKG + NADH
15	CK	ADP + PCr = ATP + Cr
16	CS	ACCOA + OXA => CIT
17	FUM	FUM => MAL

Table S1. Reaction equations (continued)

Reaction number	Reaction name	Reaction
18	G6PDH	G6P + 2 NADP => R5P + 2 NADPH
19	GLDH	GLU + NAD = AKG + NADH + NH4
20	GLN	GLN = NH4 + GLU
21	GLNt	EGLN + ATP => GLN + ADP
22	GLUt	GLU + ADP => EGLU + ATP
23	GLYt	EGLY + ATP => GLY + ADP
24	growth	0.06 EALA + 0.04 EARG + 0.03 EASN + 0.04 EASP + 0.02 ECYS + 0.03 EGLN + 0.04 EGLU + 0.06 EGLY + 0.02 EHIS + 0.03 EILE + 0.06 ELEU + 0.06 ELYS + 0.01 EMET + 0.02 EPHE + 0.03 EPRO + 0.05 ESER + 0.04 ETHR + 0.005 ETRP + 0.02 ETYR + 0.04 EVAL + 3.78 ATP + 0.03 G6P + 0.03 R5P + 0.09 CIT => X + 3.78 ADP
25	HISARGTA	EHIS + EARG + AKG => 4 GLU + NH4
26	HK	EGLC + ATP => G6P + ADP
27	IDO	ETRP => KYN
28	iNOS	ARG + NADP => CTR + NADPH + NO
29	KOT	KYN + AKG => KYT + GLU
30	LDH	PYR + NADH => ELAC + NAD
31	leak	2 NADH => 2 NAD
32	ME	MAL + NAD => PYR + NADH
33	MLD	MAL + NAD => OXA + NADH
34	NADPHox	NADPH => NADP
35	NAT	2 R5P + 2 ATP + GLN => NAD + AMP + GLU
36	NHG	NAD + ATP => NADP + ADP
37	OCT	ORN => CTR
38	PC	PYR + ATP => OXA + ADP

Table S1. Reaction equations (continued)

Reaction number	Reaction name	Reaction
39		PYR + NAD => ACCOA + NADH
40	PFK	F6P + ATP => 2 GAP + ADP
41	PGI	G6P = F6P
42	PGK	GAP + ADP + NAD => PEP + ATP + NADH
43	PK	PEP + ADP => PYR + ATP
44	PPRibP	R5P + 2 GLN + ASP + GLY + 5 ATP => 2 GLU + FUM + 4 ADP + 2 AMP
45	resp	2 NADH + 4 ADP => 2 NAD + 4 ATP
46	SCOAS	SCOA + ADP => ATP + SUC
47	SDH	SUC + 0.66 NAD + ADP => FUM + 0.66 NADH + ATP
48	SDHH	ESER => PYR + NH4
49	TK	3 R5P => 2 F6P + GAP

Table S2. Successive steps for model calibration

Step number	Used metabolite	Determined reactions	Step number	Used metabolite	Determined reactions
1	X	growth	24	ASN	ASN
2	PCr	CK	25	ASP	ASTA
3	EGLC	HK	26	ACCOA	CS & PDH
4	ELAC	LDH	27	CIT	CITS
5	ETRP	IDO	28	AKG	AKGDH & GLD
6	KYN	KOT	29	SCOA	SCOAS
7	ELEU	AAtoSUC	30	SUC	SDH
8	EHIS	HISARGTA	31	NH4	GLN
9	EGLY	GLYt	32	FUM	FUM
10	UREA	ARG1	33	GLN	NAT
11	NO	iNOS	34	AMP	AK
12	R5P	PPRibP	35	G6P	G6PDH & PGI
13	R5P	NAT.	36	NADPH	NADPHox
14	EARG	ARGt	37	NADP	NHG
15	EGLU	GLUt	38	F6P	PFK & TK
16	EGLN	GLNt	39	GAP	PGK
17	EALA	ALATA	40	PEP	PK
18	ESER	SDHH	41	R5P	EP
19	EASN	ASNt	42	MAL	ME & MLD
20	EASP	ASPt	43	OXA	PC
21	ORN	OCT	44	ADP	ATPase & resp
22	CTR	ASS	45	NAD	leak
23	AS·ARG	ASL			

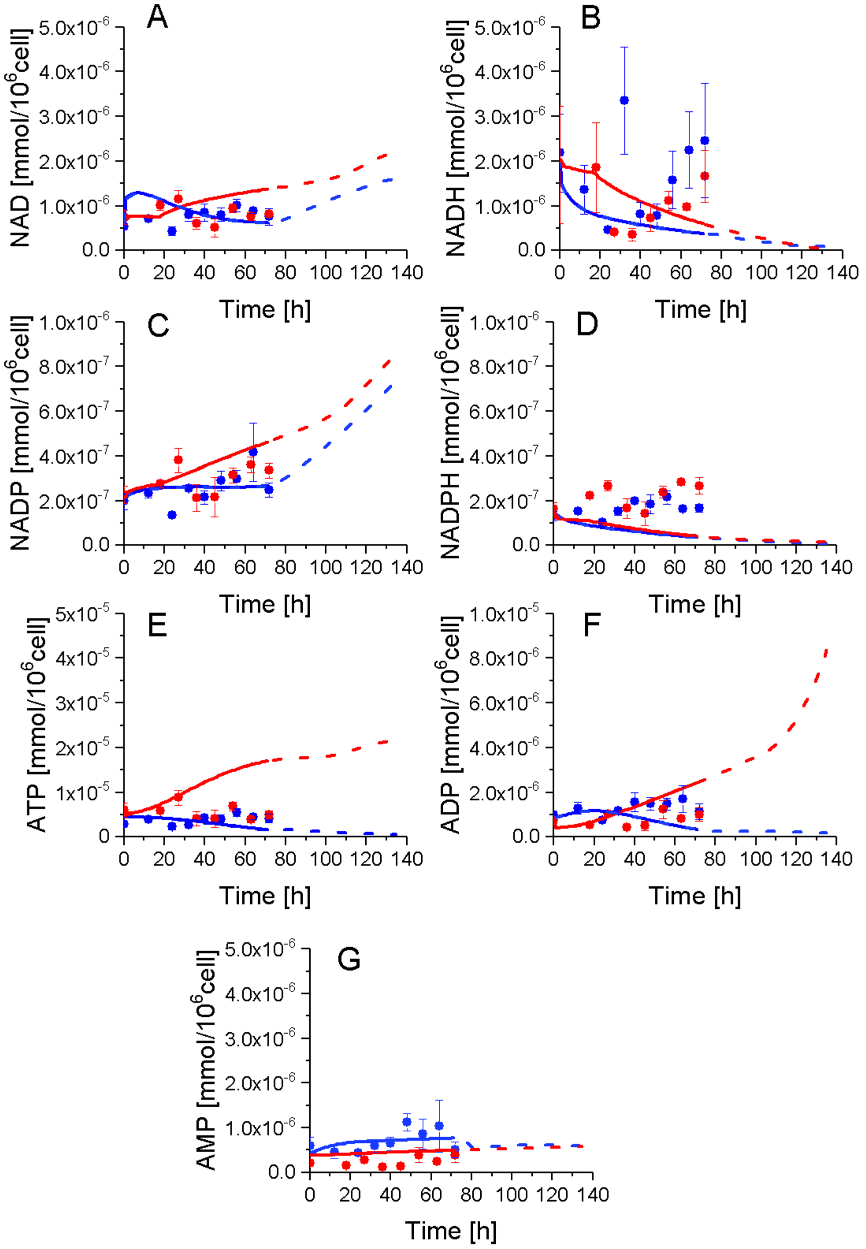

Figure S1. Experimental data and model simulation of cofactors and nucleotides concentrations· (A) Nicotinamide adenine dinucleotide concentration; (B) Nicotinamide adenine dinucleotide H concentration; (C) Nicotinamide adenine dinucleotide phosphate concentration; (D) Nicotinamide adenine dinucleotide phosphate H concentration; (E) Adenosine triphosphate concentration; (F) Adenosine diphosphate concentration; (G) Adenosine monophosphate concentration. ■ are P4 cells experimental data, ● are P9 cells experimental· Data Average values are shown for both passages (n=3). Blue line is P4 cells simulation, red line is P9 cells simulation.

Table S3. Parameter values for average simulation

Parameters	Values	Units	Parameters	Values	Units
$vmax_{AAtoSUC}$	6.20 x 10^{-04}	mmol·10^{-6}cells·h^{-1}	$km_{growth_{EPHE}}$	1.60 x 10^{-02}	mM
$vmax_{AK}$	1.92 x 10^{-04}	mmol·10^{-6}cells·h^{-1}	$km_{growth_{EPRO}}$	1.00 x 10^{-02}	mM
$vmax_{AKGDH}$	1.53 x 10^{-03}	mmol·10^{-6}cells·h^{-1}	$km_{growth_{ESER}}$	1.10 x 10^{-03}	mM
$vmax_{ALATA}$	1.09 x 10^{-04}	mmol·10^{-6}cells·h^{-1}	$km_{growth_{ETHR}}$	9.60 x 10^{-03}	mM
$vmax_{ARG1}$	1.88 x 10^{-04}	mmol·10^{-6}cells·h^{-1}	$Km_{growth_{ETYR}}$	1.20 x 10^{-02}	mM
$vmax_{ARGt}$	5.15 x 10^{-05}	mmol·10^{-6}cells·h^{-1}	$km_{growth_{EVAL}}$	1.30 x 10^{-02}	mM
$vmax_{ASL}$	1.33 x 10^{-04}	mmol·10^{-6}cells·h^{-1}	$km_{growth_{G6P}}$	9.90 x 10^{-13}	mmol·10^{-6}cells
$vmax_{ASN}$	5.00 x 10^{-06}	mmol·10^{-6}cells·h^{-1}	$km_{growth_{R5P}}$	5.82 x 10^{-12}	mmol·10^{-6}cells
$vmax_{ASNt}$	5.47 x 10^{-06}	mmol·10^{-6}cells·h^{-1}	$km_{HISARGTA_{AKG}}$	5.49 x 10^{-06}	mmol·10^{-6}cells
$vmax_{ASPt}$	9.29 x 10^{-12}	mmol·10^{-6}cells·h^{-1}	$km_{HISARGTA_{EARG}}$	4.00 x 10^{-01}	mM
$vmax_{ASS}$	1.20 x 10^{-03}	mmol·10^{-6}cells·h^{-1}	$km_{HISARGTA_{EHIS}}$	1.50 x 10^{-01}	mM
$vmax_{ASTA}$	8.90 x 10^{-07}	mmol·10^{-6}cells·h^{-1}	$km_{HK_{ATP}}$	6.00 x 10^{-05}	/
$vmax_{ATPase}$	6.64 x 10^{-03}	mmol·10^{-6}cells·h^{-1}	$km_{HK_{EGLC}}$	5.00	mM
$vmax_{CITS}$	1.08 x 10^{-03}	mmol·10^{-6}cells·h^{-1}	$ki_{HK_{G6P}}$	1.00 x 10^{-07}	mmol·10^{-6}cells
$vmax_{CK}$	2.04 x 10^{-06}	mmol·10^{-6}cells·h^{-1}	$ka_{HK_{AMP_{ATP}}}$	1.00 x 10^{-02}	/

Table S3. Parameter values for average simulation (continued)

Parameters	Values	Units	Parameters	Values	Units
$vmax_{CS}$	1.08×10^{-03}	mmol·10^{-6}cells·h^{-1}	$alpha_{HK_{AMP_{ATP}}}$	1.10	/
$vmax_{FUM}$	1.43×10^{-03}	mmol·10^{-6}cells·h^{-1}	$beta_{HK_{AMP_{ATP}}}$	1.05	/
$vmax_{G6PDH}$	5.00×10^{-05}	mmol·10^{-6}cells·h^{-1}	$km_{IDO_{ETRP}}$	1.00×10^{-02}	mM
$vmax_{GLDH}$	6.25×10^{-08}	mmol·10^{-6}cells·h^{-1}	$km_{iNOS_{ARG}}$	1.00×10^{-03}	mmol·10^{-6}cells
$vmax_{GLN}$	1.07×10^{-01}	mmol·10^{-6}cells·h^{-1}	$km_{iNOS_{NADP}}$	3.00×10^{-07}	/
$vmax_{GLNt}$	2.28×10^{-04}	mmol·10^{-6}cells·h^{-1}	$km_{KOT_{KYN}}$	1.00×10^{-02}	mM
$vmax_{GLUt}$	3.15×10^{-05}	mmol·10^{-6}cells·h^{-1}	$km_{KOT_{AKG}}$	1.00×10^{-07}	mmol·10^{-6}cells
$vmax_{GLYt}$	1.59×10^{-06}	mmol·10^{-6}cells·h^{-1}	$k\mathrm{m}_{LDH_{NADH}}$	1.01×10^{-07}	/
$vmax_{growth}$	6.97×10^{-02}	h^{-1}	$km_{LDH_{PYR}}$	1.08×10^{-07}	mmol·10^{-6}cells
$vmax_{HISARGTA}$	1.90×10^{-05}	mmol·10^{-6}cells·h^{-1}	$ki_{LDH_{PYR}}$	4.50×10^{-07}	mmol·10^{-6}cells
$vmax_{HK}$	1.00×10^{-03}	mmol·10^{-6}cells·h^{-1}	$ka_{LDH_{AMP_{ATP}}}$	9.00×10^{-02}	/
$vmax_{IDO}$	1.35×10^{-05}	mmol·10^{-6}cells·h^{-1}	$alpha_{LDH_{AMP_{ATP}}}$	4.65×10^{-01}	/
$vma\mathrm{x}_{iNOS}$	1.21×10^{-06}	mmol·10^{-6}cells·h^{-1}	$beta_{LDH_{AMP_{ATP}}}$	$1.20 \times 10^{+01}$	/
$vmax_{KOT}$	1.11×10^{-05}	mmol·10^{-6}cells·h^{-1}	$km_{leak_{NADH}}$	1.00×10^{-06}	/
$vmax_{LDH}$	8.80×10^{-05}	mmol·10^{-6}cells·h^{-1}	$km_{ME_{NAD}}$	1.00×10^{-06}	/
$vmax_{leak}$	2.39×10^{-03}	mmol·10^{-6}cells·h^{-1}	$km_{ME_{MAL}}$	1.00×10^{-06}	mmol·10^{-6}cells

Table S3. Parameter values for average simulation (continued)

Parameters	Values	Units	Parameters	Values	Units
$vmax_{ME}$	1.00 x 10^{-06}	mmol·10^{-6}cells·h^{-1}	$km_{MLD_{MAL}}$	1.00 x 10^{-06}	mmol·10^{-6}cells
$vmax_{MLD}$	1.53 x 10^{-03}	mmol·10^{-6}cells·h^{-1}	$km_{MLD_{NAD}}$	1.00 x 10^{-06}	/
$vmax_{NADPHox}$	5.33 x 10^{-05}	mmol·10^{-6}cells·h^{-1}	$km_{NADPHox_{NADPH}}$	1.00 x 10^{-07}	/
$vmax_{NAT}$	5.00 x 10^{-08}	mmol·10^{-6}cells·h^{-1}	$km_{NAT_{R5P}}$	1.00 x 10^{-10}	mmol·10^{-6}cells
$vmax_{NHG}$	1.40 x 10^{-08}	mmol·10^{-6}cells·h^{-1}	$km_{NAT_{ATP}}$	5.00 x 10^{-06}	/
$vmax_{OCT}$	1.42 x 10^{-04}	mmol·10^{-6}cells·h^{-1}	$km_{NAT_{GLN}}$	4.00 x 10^{-03}	mmol·10^{-6}cells
$vmax_{PC}$	7.90 x 10^{-07}	mmol·10^{-6}cells·h^{-1}	$km_{NHG_{NAD}}$	1.00 x 10^{-06}	mmol·10^{-6}cells
$vmax_{PDH}$	1.17 x 10^{-03}	mmol·10^{-6}cells·h^{-1}	$km_{NHG_{ATP}}$	5.00 x 10^{-06}	/
$vmax_{PFK}$	2.12 x 10^{-04}	mmol·10^{-6}cells·h^{-1}	$km_{OCT_{ORN}}$	5.01 x 10^{-02}	mmol·10^{-6}cells
$vmax_{PGI}$	9.73 x 10^{-02}	mmol·10^{-6}cells·h^{-1}	$km_{PC_{ATP}}$	5.00 x 10^{-06}	/
$vmax_{PGK}$	2.80 x 10^{-02}	mmol·10^{-6}cells·h^{-1}	$km_{PC_{PYR}}$	1.00 x 10^{-07}	mmol·10^{-6}cells
$vmax_{PK}$	9.51 x 10^{-04}	mmol·10^{-6}cells·h^{-1}	$km_{PDH_{NAD}}$	1.00 x 10^{-08}	/
$vmax_{PPRibP}$	2.40 x 10^{01}	mmol·10^{-6}cells·h^{-1}	$km_{PDH_{PYR}}$	2.00 x 10^{-07}	mmol·10^{-6}cells
$vmax_{resp}$	7.20 x 10^{-04}	mmol·10^{-6}cells·h^{-1}	$km_{PFK_{ATP}}$	3.58 x 10^{-09}	/
$vmax_{SCOAS}$	1.42 x 10^{-03}	mmol·10^{-6}cells·h^{-1}	$km_{PFK_{F6P}}$	3.00 x 10^{-07}	mmol·10^{-6}cells
$vmax_{SDH}$	1.39 x 10^{-03}	mmol·10^{-6}cells·h^{-1}	$ka_{PFK_{AMP_{ATP}}}$	9.00 x 10^{-02}	/

Table S3. Parameter values for average simulation (continued)

Parameters	Values	Units	Parameters	Values	Units
$vmax_{SDHH}$	1.21 x 10^{-05}	mmol·10^{-6}cells·h^{-1}	$alpha_{PFK_{AMP_{ATP}}}$	4.70 x 10^{-01}	/
$vmax_{TK}$	1.98 x 10^{-06}	mmol·10^{-6}cells·h^{-1}	$beta_{PFK_{AMP_{ATP}}}$	1.05 x 10^{+01}	/
$vmaxr_{AK}$	1.93 x 10^{-05}	mmol·10^{-6}cells·h^{-1}	$km_{PGI_{F6P}}$	2.01 x 10^{-05}	mmol·10^{-6}cells
$vmaxr_{ASTA}$	3.16 x 10^{-04}	mmol·10^{-6}cells·h^{-1}	$km_{PGI_{G6P}}$	1.32 x 10^{-06}	mmol·10^{-6}cells
$vmaxr_{ASN}$	1.00 x 10^{-08}	mmol·10^{-6}cells·h^{-1}	$ki_{PGI_{PEP}}$	3.70 x 10^{-07}	mmol·10^{-6}cells
$vmaxr_{CK}$	1.97 x 10^{-06}	mmol·10^{-6}cells·h^{-1}	$km_{PGK_{ADP}}$	2.51 x 10^{-08}	/
$vmaxr_{PGI}$	1.97 x 10^{-06}	mmol·10^{-6}cells·h^{-1}	$km_{PGK_{GAP}}$	1.02 x 10^{-06}	mmol·10^{-6}cells
$vmaxr_{ALATA}$	6.07 x 10^{-05}	mmol·10^{-6}cells·h^{-1}	$km_{PGK_{NAD}}$	2.21 x 10^{-08}	/
$vmaxr_{GLN}$	1.79 x 10^{-01}	mmol·10^{-6}cells·h^{-1}	$km_{PK_{ADP}}$	1.47 x 10^{-04}	/
$vmaxr_{GLDH}$	5.22 x 10^{-05}	mmol·10^{-6}cells·h^{-1}	$km_{PK_{PEP}}$	7.92 x 10^{-08}	mmol·10^{-6}cells
$v_{growth_{ATP}}$	1.19 x 10^{-02}	mmol·10^{-6}cells	$ka_{PK_{F6P}}$	2.09 x 10^{-08}	mmol·10^{-6}cells
$v_{growth_{ADP}}$	1.19 x 10^{-02}	mmol·10^{-6}cells	$alpha_{PK_{F6P}}$	9.89 x 10^{-01}	mmol·10^{-6}cells
$v_{growth_{CIT}}$	2.73 x 10^{-04}	mmol·10^{-6}cells	$beta_{PK_{F6P}}$	1.89	mmol·10^{-6}cells
$v_{growth_{EALA}}$	1.89 x 10^{-04}	mmol·10^{-6}cells	$km_{PPRibP_{ASP}}$	5.00 x 10^{-01}	mmol·10^{-6}cells
$v_{growth_{EARG}}$	1.19 x 10^{-04}	mmol·10^{-6}cells	$km_{PPRibP_{ATP}}$	5.00 x 10^{-06}	/
$v_{growth_{EASN}}$	9.07 x 10^{-05}	mmol·10^{-6}cells	$km_{PPRibP_{GLN}}$	3.00	mmol·10^{-6}cells

Table S3. Parameter values for average simulation (continued)

Parameters	Values	Units	Parameters	Values	Units
$v_{growth_{EASP}}$	1.13 x 10^{-04}	mmol·10^{-6}cells	$km_{PPRibP_{GLY}}$	2.00 x 10^{-01}	mmol·10^{-6}cells
$v_{growth_{ECYS}}$	4.57 x 10^{-05}	mmol·10^{-6}cells	$km_{PPRibP_{R5P}}$	1.00 x 10^{-11}	mmol·10^{-6}cells
$v_{growth_{EGLN}}$	1.01 x 10^{-04}	mmol·10^{-6}cells	$km_{resp_{ADP}}$	1.00 x 10^{-06}	mmol·10^{-6}cells
$v_{growth_{EGLU}}$	1.22 x 10^{-04}	mmol·10^{-6}cells	$km_{resp_{NADH}}$	2.00 x 10^{-06}	/
$v_{growth_{EGLY}}$	1.69 x 10^{-04}	mmol·10^{-6}cells	$km_{SCOAS_{ADP}}$	1.00 x 10^{-06}	/
$v_{growth_{EHIS}}$	4.50 x 10^{-05}	mmol·10^{-6}cells	$km_{SCOAS_{SCOA}}$	4.00 x 10^{-07}	mmol·10^{-6}cells
$v_{growth_{EILE}}$	1.02 x 10^{-04}	mmol·10^{-6}cells	$km_{SDH_{ADP}}$	1.00 x 10^{-06}	/
$v_{growth_{ELEU}}$	1.78 x 10^{-04}	mmol·10^{-6}cells	$km_{SDH_{NAD}}$	1.00 x 10^{-06}	mM
$v_{growth_{ELYS}}$	1.80 x 10^{-04}	mmol·10^{-6}cells	$km_{SDH_{SUC}}$	3.00 x 10^{-07}	mmol·10^{-6}cells
$v_{growth_{EMET}}$	4.35 x 10^{-05}	mmol·10^{-6}cells	$km_{SDHH_{ESER}}$	1.00 x 10^{-01}	mM
$v_{growth_{EPHE}}$	6.90 x 10^{-05}	mmol·10^{-6}cells	$km_{TK_{R5P}}$	1.09 x 10^{-07}	mmol·10^{-6}cells
$v_{growth_{EPRO}}$	9.86 x 10^{-05}	mmol·10^{-6}cells			
$v_{growth_{ESER}}$	1.35 x 10^{-04}	mmol·10^{-6}cells			
$v_{growth_{ETHR}}$	1.22 x 10^{-04}	mmol·10^{-6}cells			
$v_{growth_{ETRP}}$	1.39 x 10^{-05}	mmol·10^{-6}cells			
$v_{growth_{ETYR}}$	5.73 x 10^{-05}	mmol·10^{-6}cells			

Table S3. Parameter values for average simulation (continued)

Parameters	Values	Units
$v_{growth_{EVAL}}$	1.31 x 10^{-04}	mmol·10-6cells
$v_{growth_{G6P}}$	8.79 x 10^{-05}	mmol·10^{-6}cells
$v_{growth_{R5P}}$	7.34 x 10^{-05}	mM

Table S4. MRM transition and retention time of each amino acid

Compound name	IS	Precursor ion	Product ion	frag	CE	polarity	RT min
Cystine	NO	241.3	241	76	0	pos	14.78
Homoarginine	YES	189.2	144	92	12	pos	14.44
Tyrosine	NO	182.2	136.1	66	8	pos	8.74
Homophenylalanine	YES	180.2	134.1	75	8	pos	6.12
Arginine	NO	175.2	70.1	95	24	pos	15.09
Phenylalanine	NO	166.2	120.1	72	8	pos	6.92
Histidine	NO	156.2	110.1	81	12	pos	16.84
Methionine-d3	YES	153.2	136.1	69	4	pos	8.22
Methionine	NO	150.2	133	63	4	pos	8.18
Glutamic acid	NO	148.1	84.1	72	16	pos	12.59
Lysine	NO	147.2	84.1	66	12	pos	15.48
Glutamine	NO	147.2	84.1	72	16	pos	11.3
Aspartic acid	NO	134.1	74.1	61	12	pos	14.23
Asparagine	NO	133.1	74	60	12	pos	11.87
Isoleucine	NO	132.2	86.1	63	4	pos	7.431
Leucine	NO	132.2	86.1	72	4	pos	7.431
Threonine	NO	120.1	103.1	133	16	pos	6.94
Valine	NO	118.2	72.1	55	8	pos	9
Proline	NO	116.1	70.1	75	12	pos	9.6
Serine	NO	106.1	60.1	60	8	pos	11.62
Alanine	NO	90.1	44.1	42	8	pos	10.7
Glycine	NO	76.1	30.1	39	4	pos	11.47

Table S5. Flux kinetic equations

Number	Flux
1	$V_{AAtoSUC} = vmax_{AAtoSUC} \bullet \frac{[AKG]}{[AKG] + km_{AAtoSUC_{AKG}}} \bullet \frac{\frac{[ATP]}{[ADP]}}{\frac{[ATP]}{[ADP]} + km_{AAtoSUC_{ATP}}} \bullet \frac{\frac{[NAD]}{[NADH]}}{\frac{[NAD]}{[NADH]} + km_{AAtoSUC_{NAD}}} \bullet \frac{\frac{[NADP]}{[NADPH]}}{\frac{[NADP]}{[NADPH]} + km_{AAtoSUC_{NADP}}} \bullet \frac{[EILE]}{[EILE] + km_{AAtoSUC_{EILE}}} \bullet \frac{[EVAL]}{[EVAL] + km_{AAtoSUC_{EVAL}}} \bullet \frac{[ELEU]}{[ELEU] + km_{AAtoSUC_{ELEU}}} \bullet \frac{[ETYR]}{[ETYR] + km_{AAtoSUC_{ETYR}}} \bullet \frac{[ELYS]}{[ELYS] + km_{AAtoSUC_{ELYS}}}$
2	$V_{AK} = vmax_{AK} \bullet \frac{[AMP]}{[AMP] + km_{AK_{AMP}}} \bullet \frac{\frac{[ATP]}{[ADP]}}{\frac{[ATP]}{[ADP]} + km_{AK_{ATP}}} - vmaxr_{AK} \bullet \frac{\frac{[ADP]}{[ATP]}}{\frac{[ADP]}{[ATP]} + km_{AK_{ADP}}}$
3	$V_{AKGDH} = vmax_{AKGDH} \bullet \frac{[AKG]}{[AKG] + km_{AKGDH_{AKG}}} \bullet \frac{\frac{[NAD]}{[NADH]}}{\frac{[NAD]}{[NADH]} + km_{AKGDH_{NAD}}}$
4	$V_{ALATA} = vmax_{ALATA} \bullet \frac{[GLU]}{[GLU] + km_{ALATA_{GLU}}} \bullet \frac{[PYR]}{[PYR] + km_{ALATA_{PYR}}} - vmaxr\_ALATA \bullet \frac{[AKG]}{[AKG] + km_{ALATA_{AKG}}} \bullet \frac{[EALA]}{[EALA] + km_{ALATA_{EALA}}}$
5	$V_{ARG1} = vmax_{ARG1} \bullet \frac{[ARG]}{[ARG] + km_{ARG1_{ARG}}}$

Table S5. Flux kinetic equations (continued)

Number	Flux
6	$V_{ARGt} = vmax_{ARGt} \bullet \frac{\frac{[ATP]}{[ADP]}}{\frac{[ATP]}{[ADP]} + km_{ARGt_{ATP}}} \bullet \frac{[EARG]}{[EARG] + km_{ARGt_{EARG}}}$
7	$V_{ASL} = vmax_{ASL} \bullet \frac{[AS]}{[AS] + km_{ASL_{AS}}}$
8	$V_{ASN} = vmax_{ASN} \bullet \frac{[ASN]}{[ASN] + km_{ASN_{ASN}}} - vmaxr_{ASN} \bullet \frac{[ASP]}{[ASP] + km_{ASN_{ASP}}} \bullet \frac{[NH4]}{[NH4] + km_{ASN_{NH4}}}$
9	$V_{ASNt} = vmax_{ASNt} \bullet \frac{\frac{[ATP]}{[ADP]}}{\frac{[ATP]}{[ADP]} + km_{ASNt_{ATP}}} \bullet \frac{[EASN]}{[EASN] + km_{ASNt_{EASN}}}$
10	$V_{ASPt} = vmax_{ASPt} \bullet \frac{\frac{[ATP]}{[ADP]}}{\frac{[ATP]}{[ADP]} + km_{ASPt_{ATP}}} \bullet \frac{[EASP]}{[EASP] + km_{ASPt_{EASP}}}$
11	$V_{ASS} = vmax_{ASS} \bullet \frac{[ASP]}{[ASP] + km_{ASS_{ASP}}} \bullet \frac{\frac{[ATP]}{[ADP]}}{\frac{[ATP]}{[ADP]} + km_{ASS_{ATP}}} \bullet \frac{[CTR]}{[CTR] + km_{ASS_{CTR}}}$

Table S5. Flux kinetic equations (continued)

Number	Flux
12	$V_{ASTA} = vmax_{ASTA} \bullet \frac{[AKG]}{[AKG] + km_{ASTA_{AKG}}} \bullet \frac{[ASP]}{[ASP] + km_{ASTA_{ASP}}} - vmaxr_{ASTA} \bullet \frac{[GLU]}{[GLU] + km_{ASTA_{GLU}}} \bullet \frac{[OXA]}{[OXA] + km_{ASTA_{OXA}}}$
13	$V_{ATPase} = vmax_{ATPase} * \frac{[ATP]}{[ATP] + km_{ATPase_{ATP}}}$
14	$V_{CITS} = vmax_{CITS} \bullet \frac{[CIT]}{[CIT] + km_{CITS_{CIT}}} \bullet \frac{\frac{[NAD]}{[NADH]}}{\frac{[NAD]}{[NADH]} + km_{CITS_{NAD}}}$
15	$V_{CK} = vmax_{CK} \bullet \frac{\frac{[ADP]}{[ATP]}}{\frac{[ADP]}{[ATP]} + km_{CK_{ADP}}} \bullet \frac{[PCr]}{[PCr] + km_{CK_{PCr}}} - vmaxr_{CK} \bullet \frac{\frac{[ATP]}{[ADP]}}{\frac{[ATP]}{[ADP]} + km_{CK_{ATP}}} \bullet \frac{[Cr]}{[Cr] + km_{CK_{Cr}}}$
16	$V_{CS} = vmax_{CS} \bullet \frac{[ACCOA]}{[ACCOA] + km_{CS_{ACCOA}}} \bullet \frac{[OXA]}{[OXA] + km_{CS_{OXA}}}$
17	$V_{FUM} = vmax_{FUM} \bullet \frac{[FUM]}{[FUM] + km_{FUM_{FUM}}}$

Table S5. Flux kinetic equations (continued)

Number	Flux
18	$V_{G6PDH} = vmax_{G6PDH} \bullet \frac{[G6P]}{[G6P] + km_{G6PDH_{G6P}}} \bullet \frac{\frac{[NADP]}{[NADPH]}}{\frac{[NADP]}{[NADPH]} + km_{G6PDH_{NADP}}}$
19	$V_{GLDH} = vmax_{GLDH} \bullet \frac{[GLU]}{[GLU] + km_{GLDH_{GLU}}} \bullet \frac{\frac{[NAD]}{[NADH]}}{\frac{[NAD]}{[NADH]} + km_{GLDH_{NAD}}} - vmaxr_{GLDH} \bullet \frac{[AKG]}{[AKG] + km_{GLDH_{AKG}}} \bullet \frac{\frac{[NADH]}{[NAD]}}{\frac{[NADH]}{[NAD]} + km_{GLDH_{NADH}}} \bullet \frac{[NH4]}{[NH4] + km_{GLDH_{NH4}}}$
20	$V_{GLN} = vmax_{GLN} \bullet \frac{[GLN]}{[GLN] + km_{GLN_{GLN}}} - vmaxr\_GLN \bullet \frac{[GLU]}{[GLU] + km_{GLN_{GLU}}} \bullet \frac{[NH4]}{[NH4] + km_{GLN_{NH4}}}$
21	$V_{GLNt} = vmax_{GLNt} \bullet \frac{\frac{[ATP]}{[ADP]}}{\frac{[ATP]}{[ADP]} + km_{GLNt_{ATP}}} \bullet \frac{[EGLN]}{[EGLN] + km_{GLNt_{EGLN}}}$
22	$V_{GLUt} = vmax_{GLUt} * \frac{\frac{[ADP]}{[ATP]}}{\frac{[ADP]}{[ATP]} + km_{GLUt_{ADP}}} * \frac{[GLU]}{[GLU] + km_{GLUt_{GLU}}}$

Table S5. Flux kinetic equations (continued)

Number	Flux
23	$V_{GLYt} = vmax_{GLYt} * \frac{\frac{[ATP]}{[ADP]}}{\frac{[ATP]}{[ADP]} + km_{GLYt_{ATP}}} * \frac{[EGLY]}{[EGLY] + km_{GLYt_{EGLY}}}$
24	$V_{growth} = vmax_{growth} * \frac{\frac{[ATP]}{[ADP]}}{\frac{[ATP]}{[ADP]} + km_{growth_{ATP}}} * \frac{[CIT]}{[CIT] + km_{growth_{CIT}}} * \frac{[G6P]}{[G6P] + km_{growth_{G6P}}} * \frac{[R5P]}{[R5P] + km_{growth_{R5P}}} * \frac{[EARG]}{[EARG] + km_{growth_{EARG}}} * \frac{[ETRP]}{[ETRP] + km_{growth_{ETRP}}} * \frac{[EGLU]}{[EGLU] + km_{growth_{EGLU}}} * \frac{[EGLN]}{[EGLN] + km_{growth_{EGLN}}} * \frac{[EALA]}{[EALA] + km_{growth_{EALA}}} * \frac{[ESER]}{[ESER] + km_{growth_{ESER}}} * \frac{[EASN]}{[EASN] + km_{growth_{EASN}}} * \frac{[EASP]}{[EASP] + km_{growth_{EASP}}} * \frac{[EILE]}{[EILE] + km_{growth_{EILE}}} * \frac{[EVAL]}{[EVAL] + km_{growth_{EVAL}}} * \frac{[ETHR]}{[ETHR] + km_{growth_{ETHR}}} * \frac{[ELEU]}{[ELEU] + km_{growth_{ELEU}}} * \frac{[ETYR]}{[ETYR] + km_{growth_{ETYR}}} * \frac{[ECYS]}{[ECYS] + km_{growth_{ECYS}}} * \frac{[ELYS]}{[ELYS] + km_{growth_{ELYS}}} * \frac{[EPRO]}{[EPRO] + km_{growth_{EPRO}}} * \frac{[EMET]}{[EMET] + km_{growth_{EMET}}} * \frac{[EPHE]}{[EPHE] + km_{growth_{EPHE}}} * \frac{[EHIS]}{[EHIS] + km_{growth_{EHIS}}} * \frac{[EGLY]}{[EGLY] + km_{growth_{EGLY}}}$

Table S5. Flux kinetic equations (continued)

Number	Flux
25	$V_{HISARGTA} = vmax_{HISARGTA} * \frac{[AKG]}{[AKG] + km_{HISARGTA_{AKG}}} * \frac{[EARG]}{[EARG] + km_{HISARGTA_{EARG}}} * \frac{[EHIS]}{[EHIS] + km_{HISARGTA_{EHIS}}}$
26	$V_{HK} = vmax_{HK} * \frac{\frac{[ATP]}{[ADP]}}{\frac{[ATP]}{[ADP]} + km_{HK_{ATP}}} * \frac{[EGLC] * \left(1 + \frac{beta_{HK_{AMP_{ATP}}} * \frac{[AMP]}{[ATP]}}{alpha_{HK_{AMP_{ATP}}} * ka_{HK_{AMP_{ATP}}}}\right)}{km_{HK_{EGLC}} * \left(1 + \frac{\frac{[AMP]}{[ATP]}}{ka_{HK_{AMP_{ATP}}}}\right) + [EGLC] * \left(1 + \frac{\frac{[AMP]}{[ATP]}}{alpha_{HK_{AMP_{ATP}}} * ka_{HK_{AMP_{ATP}}}}\right)} * \frac{ki_{HK_{G6P}}}{ki_{HK_{G6P}} + [G6P]}$
27	$V_{IDO} = vmax_{IDO} * \frac{[ETRP]}{[ETRP] + km_{IDO_{ETRP}}}$
28	$V_{iNOS} = vmax_{iNOS} * \frac{[ARG]}{[ARG] + km_{iNOS_{ARG}}} * \frac{\frac{[NADP]}{[NADPH]}}{\frac{[NADP]}{[NADPH]} + km_{iNOS_{NADP}}}$

Table S5. Flux kinetic equations (continued)

Number	Flux
29	$V_{KOT} = vmax_{KOT} * \frac{[AKG]}{[AKG] + km_{KOT_{AKG}}} * \frac{[KYN]}{[KYN] + km_{KOT_{KYN}}}$
30	$V_{LDH} = vmax_{LDH} * \frac{\frac{[NADH]}{[NAD]}}{\frac{[NADH]}{[NAD]} + km_{LDH_{NADH}}} * \frac{[PYR] * \left(1 + \frac{beta_{LDH_{AMP_{ATP}}} * \frac{[AMP]}{[ATP]}}{alpha_{LDH_{AMP_{ATP}}} * ka_{LDH_{AMP_{ATP}}}}\right)}{km_{LDH_{PYR}} * \left(1 + \frac{\frac{[AMP]}{[ATP]}}{ka_{LDH_{AMP_{ATP}}}}\right) + [PYR] * \left(1 + \frac{\frac{[AMP]}{[ATP]}}{alpha_{LDH_{AMP_{ATP}}} * ka_{LDH_{AMP_{ATP}}}}\right)}$
31	$V_{leak} = vmax_{leak} * \frac{[NADH]}{[NADH] + km_{leak_{NADH}}}$
32	$V_{ME} = vmax_{ME} * \frac{[MAL]}{[MAL] + km_{ME_{MAL}}} * \frac{\frac{[NAD]}{[NADH]}}{\frac{[NAD]}{[NADH]} + km_{ME_{NAD}}}$
33	$V_{MLD} = vmax_{MLD} * \frac{[MAL]}{[MAL] + km_{MLD_{MAL}}} * \frac{\frac{[NAD]}{[NADH]}}{\frac{[NAD]}{[NADH]} + km_{MLD_{NAD}}}$

Table S5. Flux kinetic equations (continued)

Number	Flux
34	$V_{NADPHox} = vmax_{NADPHox} * \frac{[NADPH]}{[NADPH] + km_{NADPHox_{NADPH}}}$
35	$V_{NAT} = vmax_{NAT} * \frac{\frac{[ATP]}{[ADP]}}{\frac{[ATP]}{[ADP]} + km_{NAT_{ATP}}} * \frac{[GLN]}{[GLN] + km_{NAT_{GLN}}} * \frac{[R5P]}{[R5P] + km_{NAT_{R5P}}}$
36	$V_{NHG} = vmax_{NHG} * \frac{\frac{[ATP]}{[ADP]}}{\frac{[ATP]}{[ADP]} + km_{NHG_{ATP}}} * \frac{\frac{[NAD]}{[NADH]}}{\frac{[NAD]}{[NADH]} + km_{NHG_{NAD}}}$
37	$V_{OCT} = vmax_{OCT} * \frac{[ORN]}{[ORN] + km_{OCT_{ORN}}}$
38	$V_{PC} = vmax_{PC} * \frac{\frac{[ATP]}{[ADP]}}{\frac{[ATP]}{[ADP]} + km_{PC_{ATP}}} * \frac{[PYR]}{[PYR] + km_{PC_{PYR}}}$

Table S5. Flux kinetic equations (continued)

Number	Flux
39	$V_{PDH} = vmax_{PDH} * \frac{\frac{[NAD]}{[NADH]}}{\frac{[NAD]}{[NADH]} + km_{PDH_{NAD}}} * \frac{[PYR]}{[PYR] + km_{PDH_{PYR}}}$
40	$V_{PFK} = vmax_{PFK} * \frac{\frac{[ATP]}{[ADP]}}{\frac{[ATP]}{[ADP]} + km_{PFK_{ATP}}} * \frac{[F6P] * \left(1 + \frac{beta_{PFK_{AMP_{ATP}}} * \frac{[AMP]}{[ATP]}}{alpha_{PFK_{AMP_{ATP}}} * ka_{PFK_{AMP_{ATP}}}}\right)}{km_{PFK_{F6P}} * \left(1 + \frac{\frac{[AMP]}{[ATP]}}{ka_{PFK_{AMP_{ATP}}}}\right) + [F6P] * \left(1 + \frac{\frac{[AMP]}{[ATP]}}{alpha_{PFK_{AMP_{ATP}}} * ka_{PFK_{AMP_{ATP}}}}\right)}$
41	$V_{PGI} = vmax_{PGI} * \frac{[G6P]}{[G6P] + km_{PGI_{G6P}}} * \frac{ki_{PGI_{PEP}}}{ki_{PGI_{PEP}} + [PEP]} - vmaxr_{PGI} * \frac{[F6P]}{[F6P] + km_{PGI_{F6P}}}$
42	$V_{PGK} = vmax_{PGK} * \frac{\frac{[ADP]}{[ATP]}}{\frac{[ADP]}{[ATP]} + km_{PGK_{ADP}}} * \frac{[GAP]}{[GAP] + km_{PGK_{GAP}}} * \frac{\frac{[NAD]}{[NADH]}}{\frac{[NAD]}{[NADH]} + km_{PGK_{NAD}}}$

Table S5. Flux kinetic equations (continued)

Number	Flux
43	$V_{PK} = vmax_{PK} * \frac{\frac{[ADP]}{[ATP]}}{\frac{[ADP]}{[ATP]} + km_{PK_{ADP}}} * \frac{[PEP] * \left(1 + \frac{beta_{PK_{F6P}} * [F6P]}{alpha_{PK_{F6P}} * ka_{PK_{F6P}}}\right)}{km_{PK_{PEP}} * \left(1 + \frac{[F6P]}{ka_{PK_{F6P}}}\right) + [PEP] * \left(1 + \frac{[F6P]}{alpha_{PK_{F6P}} * ka_{PK_{F6P}}}\right)}$
44	$V_{PPRibP} = vmax_{PPRibP} * \frac{[ASP]}{[ASP] + km_{PPRibP_{ASP}}} * \frac{\frac{[ATP]}{[ADP]}}{\frac{[ATP]}{[ADP]} + km_{PPRibP_{ATP}}} * \frac{[GLN]}{[GLN] + km_{PPRibP_{GLN}}} * \frac{[GLY]}{[GLY] + km_{PPRibP_{GLY}}} * \frac{[R5P]}{[R5P] + km_{PPRibP_{R5P}}}$
45	$V_{resp} = vmax_{resp} * \frac{[ADP]}{[ADP] + km_{resp_{ADP}}} * \frac{[NADH]}{[NADH] + km_{resp_{NADH}}}$
46	$V_{SCOAS} = vmax_{SCOAS} * \frac{\frac{[ADP]}{[ATP]}}{\frac{[ADP]}{[ATP]} + km_{SCOAS_{ADP}}} * \frac{[SCOA]}{[SCOA] + km_{SCOAS_{SCOA}}}$
47	$V_{SDH} = vmax_{SDH} * \frac{\frac{[ADP]}{[ATP]}}{\frac{[ADP]}{[ATP]} + km_{SDH_{ADP}}} * \frac{\frac{[NAD]}{[NADH]}}{\frac{[NAD]}{[NADH]} + km_{SDH_{NAD}}} * \frac{[SUC]}{[SUC] + km_{SDH_{SUC}}}$

Table S5. Flux kinetic equations (continued)

Number	Flux
48	$V_{SDHH} = vmax_{SDHH} * \frac{[ESER]}{[ESER] + km_{SDHH_{ESER}}}$
49	$V_{TK} = vmax_{TK} * \frac{[R5P]}{[R5P] + km_{TK_{R5P}}}$

Table S6. Mass balances on metabolite concentrations

Equation number	Differential equations
1	$\frac{d[ACCOA]}{dt} = (8 * V_{AAtoSUC}) - V_{CS} + V_{PDH} - (V_{growth} * [ACCOA])$
2	$\frac{d[ADP]}{dt} = V_{AAtoSUC} + (2 * V_{AK}) + V_{ARGt} + V_{ASNt} + V_{ASPt} + V_{ATPase} - V_{CK} + V_{GLNt} - V_{GLUt} + V_{GLYt} + V_{HK} + V_{NHG} + V_{PC} + V_{PFK} - V_{PGK} - V_{PK} + (4 * V_{PPRibP}) - (4 * V_{resp}) - V_{SCOAS} - V_{SDH} - (V_{growth} * [ADP]) + (V_{growth_{ADP}} * V_{growth})$
3	$\frac{d[AKG]}{dt} = -(7 * V_{AAtoSUC}) - V_{AKGDH} + V_{ALATA} - V_{ASTA} + V_{CITS} + V_{GLDH} - V_{HISARGTA} - V_{KOT} - (V_{growth} * [AKG])$
4	$\frac{d[AMP]}{dt} = -V_{AK} + V_{ASS} + V_{NAT} + (2 * V_{PPRibP}) - (V_{growth} * [AMP])$
5	$\frac{d[ARG]}{dt} = -V_{ARG1} + V_{ARGt} + V_{ASL} - V_{iNOS} - (V_{growth} * [ARG])$
6	$\frac{d[AS]}{dt} = -V_{ASL} + V_{ASS} - (V_{growth} * [AS])$
7	$\frac{d[ASN]}{dt} = -V_{ASN} + V_{ASNt} - (V_{growth} * [ASN])$

Table S6. Mass balances on metabolite concentrations (continued)

Equation number	Differential equations
8	$\frac{d[ASP]}{dt} = V_{ASN} + V_{ASPt} - V_{ASS} - V_{ASTA} - V_{PPRibP} - (V_{growth} * [ASP])$
9	$\frac{d[ATP]}{dt} = -V_{AAtoSUC} - V_{AK} - V_{ARGt} - V_{ASNt} - V_{ASPt} - V_{ASS} - V_{ATPase} + V_{CK} - V_{GLNt} + V_{GLUt} - V_{GLYt} - V_{HK} - (2 * V_{NAT}) - V_{NHG} - V_{PC} - V_{PFK} + V_{PGK} + V_{PK} - (5 * V_{PPRibP}) + (4 * V_{resp}) + V_{SCOAS} + V_{SDH} - (V_{growth} * [ATP]) - (V_{growth_{ATP}} * V_{growth})$
10	$\frac{d[CIT]}{dt} = -V_{CITS} + V_{CS} - (V_{growth} * [CIT]) - (V_{growth_{CIT}} * V_{growth})$
11	$\frac{d[F6P]}{dt} = -V_{PFK} + V_{PGI} + (2 * V_{TK}) - (V_{growth} * [F6P])$
12	$\frac{d[FUM]}{dt} = V_{ASL} - V_{FUM} + V_{PPRibP} + V_{SDH} - (V_{growth} * [FUM])$
13	$\frac{d[G6P]}{dt} = -V_{G6PDH} + V_{HK} - V_{PGI} - (V_{growth} * [G6P]) - (V_{growth_{G6P}} * V_{growth})$
14	$\frac{d[GAP]}{dt} = (2 * V_{PFK}) - V_{PGK} + V_{TK} - (V_{growth} * [GAP])$
15	$\frac{d[GLN]}{dt} = -V_{GLN} + V_{GLNt} - V_{NAT} - (2 * V_{PPRibP}) - (V_{growth} * [GLN])$

Table S6. Mass balances on metabolite concentrations (continued)

Equation number	Differential equations
16	$\frac{d[GLU]}{dt} = (4 * V_{AAtoSUC}) - V_{ALATA} + V_{ASTA} - V_{GLDH} + V_{GLN} - V_{GLUt} + (4 * V_{HISARGTA}) + V_{KOT} + V_{NAT} + (2 * V_{PPRibP}) - (V_{growth} * [GLU])$
17	$d\frac{[GLY]}{dt} = V_{GLYt} - V_{PPRibP} - (V_{growth} * [GLY])$
18	$\frac{d[MAL]}{dt} = V_{AAtoSUC} + V_{FUM} - V_{ME} - V_{MLD} - (V_{growth} * [MAL])$
19	$\frac{d[NAD]}{dt} = -(9 * V_{AAtoSUC}) - V_{AKGDH} - V_{CITS} - V_{GLDH} + V_{LDH} + (2 * V_{leak}) - V_{ME} - V_{MLD} + V_{NAT} - V_{NHG} - V_{PDH} - V_{PGK} + (2 * V_{resp}) - (0.66 * V_{SDH}) - (V_{growth} * [NAD])$
20	$\frac{d[NADH]}{dt} = (9 * V_{AAtoSUC}) + V_{AKGDH} + V_{CITS} + V_{GLDH} - V_{LDH} - (2 * V_{leak}) + V_{ME} + V_{MLD} + V_{PDH} + V_{PGK} - (2 * V_{resp}) + (0.66 * V_{SDH}) - (V_{growth} * [NADH])$
21	$\frac{d[NADP]}{dt} = -(2 * V_{AAtoSUC}) - (2 * V_{G6PDH}) - V_{iNOS} + V_{NADPHox} + V_{NHG} - (V_{growth} * [NADP])$
22	$\frac{d[NADPH]}{dt} = (2 * V_{AAtoSUC}) + (2 * V_{G6PDH}) + V_{iNOS} - V_{NADPHox} - (V_{growth} * [NADPH])$
23	$\frac{d[OXA]}{dt} = V_{ASTA} - V_{CS} + V_{MLD} + V_{PC} - (V_{growth} * [OXA])$

Table S6. Mass balances on metabolite concentrations (continued)

Equation number	Differential equations
24	$\frac{d[PCr]}{dt} = -V_{CK} - (V_{growth} * [PCr])$
25	$\frac{d[PEP]}{dt} = V_{PGK} - V_{PK} - (V_{growth} * [PEP])$
26	$\frac{d[PYR]}{dt} = -V_{ALATA} - V_{LDH} + V_{ME} - V_{PC} - V_{PDH} + V_{PK} + V_{SDHH} - (V_{growth} * [PYR])$
27	$\frac{d[R5P]}{dt} = V_{G6PDH} - (2 * V_{NAT}) - V_{PPRibP} - (3 * V_{TK}) - (V_{growth} * [R5P]) - (V_{growth_{R5P}} * V_{growth})$
28	$\frac{d[SCOA]}{dt} = V_{AKGDH} - V_{SCOAS} - (V_{growth} * [SCOA])$
29	$\frac{d[SUC]}{dt} = (3 * V_{AAtoSUC}) + V_{SCOAS} - V_{SDH} - (V_{growth} * [SUC])$
30	$\frac{d[EARG]}{dt} = (-V_{ARGt} - V_{HISARGTA} - V_{growth_{EARG}} * V_{growth}) * ([X] * 1000)$
31	$\frac{d[ORN]}{dt} = (V_{ARG1} - V_{OCT}) * ([X] * 1000)$
32	$\frac{d[CTR]}{dt} = (-V_{ASS} + V_{iNOS} + V_{OCT}) * ([X] * 1000)$

Table S6. Mass balances on metabolite concentrations (continued)

Equation number	Differential equations
33	$\frac{d[EGLC]}{dt} = (-V_{HK}) * ([X] * 1000)$
34	$\frac{d[ELAC]}{dt} = (V_{LDH}) * ([X] * 1000)$
35	$\frac{d[\mathrm{ETRP}]}{\mathrm{dt}} = \left(-\mathrm{V_{IDO}} - \mathrm{V_{growth_{ETRP}}} * \mathrm{V_{growth}}\right) * ([\mathrm{X}] * 1000)$
36	$\frac{d[\mathrm{KYN}]}{\mathrm{dt}} = (\mathrm{V_{IDO}} - \mathrm{V_{KOT}}) * ([\mathrm{X}] * 1000)$
37	$\frac{d[\mathrm{KYT}]}{\mathrm{dt}} = (\mathrm{V_{KOT}}) * ([\mathrm{X}] * 1000)$
38	$\frac{d[\mathrm{EGLU}]}{\mathrm{dt}} = \left(\mathrm{V_{GLUt}} - \mathrm{V_{growth_{EGLU}}} * \mathrm{V_{growth}}\right) * ([\mathrm{X}] * 1000)$
39	$\frac{d[\mathrm{EGLN}]}{\mathrm{dt}} = \left(-\mathrm{V_{GLNt}} - \mathrm{V_{growth_{EGLN}}} * \mathrm{V_{growth}}\right) * ([\mathrm{X}] * 1000)$
40	$\frac{d[\mathrm{EALA}]}{\mathrm{dt}} = \left(\mathrm{V_{ALATA}} - \mathrm{V_{growth_{EALA}}} * \mathrm{V_{growth}}\right) * ([\mathrm{X}] * 1000)$
41	$\frac{d[\mathrm{ESER}]}{\mathrm{dt}} = \left(-\mathrm{V_{SDHH}} - \mathrm{V_{growth_{ESER}}} * \mathrm{V_{growth}}\right) * ([\mathrm{X}] * 1000)$

Table S6. Mass balances on metabolite concentrations (continued)

Equation number	Differential equations
42	$\frac{d[\mathrm{EASN}]}{\mathrm{dt}} = \left(-\mathrm{V_{ASNt}} - \mathrm{V_{growth_{EASN}}} * \mathrm{V_{growth}}\right) * ([\mathrm{X}] * 1000)$
43	$\frac{d[\mathrm{EASP}]}{\mathrm{dt}} = \left(-\mathrm{V_{ASPt}} - \mathrm{V_{growth_{EASP}}} * \mathrm{V_{growth}}\right) * ([\mathrm{X}] * 1000)$
44	$\frac{d[\mathrm{EILE}]}{\mathrm{dt}} = \left(-\mathrm{V_{AAtoSUC}} - \mathrm{V_{growth_{EILE}}} * \mathrm{V_{growth}}\right) * ([\mathrm{X}] * 1000)$
45	$\frac{d[\mathrm{EVAL}]}{\mathrm{dt}} = \left(-\mathrm{V_{AAtoSUC}} - \mathrm{V_{growth_{EVAL}}} * \mathrm{V_{growth}}\right) * ([\mathrm{X}] * 1000)$
46	$\frac{d[\mathrm{ETHR}]}{\mathrm{dt}} = \left(-\mathrm{V_{growth_{ETHR}}} * \mathrm{V_{growth}}\right) * ([\mathrm{X}] * 1000)$
47	$\frac{d[\mathrm{ELEU}]}{\mathrm{dt}} = \left(-\mathrm{V_{AAtoSUC}} - \mathrm{V_{growth_{ELEU}}} * \mathrm{V_{growth}}\right) * ([\mathrm{X}] * 1000)$
48	$\frac{d[\mathrm{ETYR}]}{\mathrm{dt}} = \left(-\mathrm{V_{AAtoSUC}} - \mathrm{V_{growth_{ETYR}}} * \mathrm{V_{growth}}\right) * ([\mathrm{X}] * 1000)$
49	$\frac{d[\mathrm{ECYS}]}{\mathrm{dt}} = \left(-\mathrm{V_{growth_{ECYS}}} * \mathrm{V_{growth}}\right) * ([\mathrm{X}] * 1000)$
50	$\frac{d[\mathrm{ELYS}]}{\mathrm{dt}} = \left(-\mathrm{V_{AAtoSUC}} - \mathrm{V_{growth_{ELYS}}} * \mathrm{V_{growth}}\right) * ([\mathrm{X}] * 1000)$

Table S6. Mass balances on metabolite concentrations (continued)

Equation number	Differential equations
51	$\frac{d[\mathrm{EPRO}]}{\mathrm{dt}} = \left(-\mathrm{V}_{\mathrm{growth}_{\mathrm{EPRO}}} * \mathrm{V}_{\mathrm{growth}}\right) * ([\mathrm{X}] * 1000)$
52	$\frac{d[\mathrm{EMET}]}{\mathrm{dt}} = \left(-\mathrm{V}_{\mathrm{growth}_{\mathrm{EMET}}} * \mathrm{V}_{\mathrm{growth}}\right) * ([\mathrm{X}] * 1000)$
53	$\frac{d[\mathrm{EPHE}]}{\mathrm{dt}} = \left(-\mathrm{V}_{\mathrm{growth}_{\mathrm{EPHE}}} * \mathrm{V}_{\mathrm{growth}}\right) * ([\mathrm{X}] * 1000)$
54	$\frac{d[\mathrm{EHIS}]}{\mathrm{dt}} = \left(-\mathrm{V}_{\mathrm{HISARGTA}} - \mathrm{V}_{\mathrm{growth}_{\mathrm{EHIS}}} * \mathrm{V}_{\mathrm{growth}}\right) * ([\mathrm{X}] * 1000)$
55	$\frac{d[\mathrm{NH4}]}{\mathrm{dt}} = (\mathrm{V}_{\mathrm{ASN}} + \mathrm{V}_{\mathrm{GLDH}} + \mathrm{V}_{\mathrm{GLN}} + \mathrm{V}_{\mathrm{HISARGTA}} + \mathrm{V}_{\mathrm{SDHH}}) * ([\mathrm{X}] * 1000)$
56	$\frac{d[\mathrm{EGLY}]}{\mathrm{dt}} = \left(-\mathrm{V}_{\mathrm{GLYt}} - \mathrm{V}_{\mathrm{growth}_{\mathrm{EGLY}}} * \mathrm{V}_{\mathrm{growth}}\right) * ([\mathrm{X}] * 1000)$
57	$\frac{d[\mathrm{UREA}]}{\mathrm{dt}} = (\mathrm{V}_{\mathrm{ARG1}}) * ([\mathrm{X}] * 1000)$
58	$\frac{d[\mathrm{X}]}{\mathrm{dt}} = \left([\mathrm{X}] * \mathrm{V}_{\mathrm{growth}}\right)$
59	$\frac{d[\mathrm{NO}]}{\mathrm{dt}} = (\mathrm{V}_{\mathrm{iNOS}}) * ([\mathrm{X}] * 1000)$
60	$\frac{d[\mathrm{Cr}]}{\mathrm{dt}} = (\mathrm{V}_{\mathrm{CK}}) * ([\mathrm{X}] * 1000)$
